# A novel Mxene-SPR-based sensor for sensing different types of cancers

**DOI:** 10.3389/fmed.2025.1608424

**Published:** 2025-08-06

**Authors:** Talia Tene, Marcelo León, Yesenia Cevallos, Paola Gabriela Vinueza-Naranjo, Deysi Inca, Said Boukerche, Cristian Vacacela Gomez

**Affiliations:** ^1^Department of Chemistry, Universidad Técnica Particular de Loja, Loja, Ecuador; ^2^Universidad Estatal Peninsula de Santa Elena, La Libertad, Ecuador; ^3^Universidad San Francisco de Quito IMNE, Diego de Robles s/n, Cumbayá, Quito, Ecuador; ^4^College of Engineering, Universidad Nacional de Chimborazo, Riobamba, Ecuador; ^5^ETEL Research Group, Faculty of Engineering and Applied Sciences, Networking and Telecommunications Engineering, Universidad de Las Américas (UDLA), Quito, Ecuador; ^6^Department of Matter Sciences, Faculty of Science and Technology, University Mohamed Cherif Messaadia of Souk Ahras, Souk Ahras, Algeria; ^7^Laboratory of Surfaces Engineering (LIS), University Badji Mokhtar of Annaba, Annaba, Algeria; ^8^INFN-Laboratori Nazionali di Frascati, Frascati, Italy; ^9^Universidad ECOTEC, Samborondón, Ecuador

**Keywords:** surface plasmon theory, cancer, Kretschmann configuration, transfer matrix method, silicon nitride, Mxene

## Abstract

Early-stage cancer screening benefits from optical transducers capable of reading minute refractive-index deviations in biofluids. This work models a surface-plasmon-resonance (SPR) biosensor that stacks copper, silicon nitride, and MXene in Kretschmann geometry and evaluates its response to six tumour-related refractive-index increments (Δn = 0.014–0.024 RIU). Transfer-matrix calculations guide a layer-by-layer optimisation: 40 nm Cu, 7 nm Si₃N₄, and two MXene sheets form the best-balanced configuration (Sys₃), while a single MXene layer on 45 nm Cu (Sys₄) offers an alternative with lower optical loss. The optimised MXene sensors raise angular sensitivity to 254° RIU^−1^ (Sys₃) and 312° RIU^−1^ (Sys₄) for the breast-T2 model, more than doubling the response of a dielectric-only stack and approaching values reported for multi-metal reference designs. Quality factors range from 48 to 58 RIU^−1^ in Sys₄ and 30 to 35 RIU^−1^ in Sys₃, corresponding detection limits fall near 2 × 10^−5^ RIU, sufficient to resolve the smallest Δn in the cancer panel. Optical loss remains below 9% in Sys₃ and under 8% in Sys₄, preserving reflected-intensity contrast for angle tracking. These results indicate that a copper platform augmented with sub-nanometre MXene and a thin Si₃N₄ spacer can match state-of-the-art sensitivity while relying on a single plasmonic metal and low-temperature fabrication. The study is purely theoretical and uses bulk refractive-index shifts as the sensing mechanism, future work should address surface chemistry, fabrication tolerances, and clinical validation.

## 1 Introduction

Global cancer incidence now exceeds 19 million new cases each year (1), and mortality remains elevated despite major advances in therapy and palliative care (2). Early detection increases the likelihood of curative intervention because lesions can be treated while still localised or when circulating tumour markers first appear (3). Diagnostic procedures differ by tumour origin: dermoscopy and histology for skin malignancies (4), Papanicolaou cytology for cervical carcinoma (5), complete blood counts with flow cytometry for haematologic neoplasms (6), endocrine panels for adrenal tumours (7), and imaging combined with biopsy for breast cancer at T1 and T2 stages (8). Many of these methods involve invasive sampling, substantial laboratory infrastructure, or ionising radiation; cost and access barriers persist, particularly in low- and middle-income regions (9).

Optical biosensors provide contact-free interrogation of biochemical events with relatively compact hardware (10). Examples that have entered oncological diagnostics include fluorescence immunoassays (11), surface-enhanced Raman scattering chips (12), photonic crystal slabs (13), and optical coherence tomography (14). Among optical transducers, surface plasmon resonance (SPR) sensors offer real-time, label-free tracking of refractive-index changes at metal–dielectric interfaces (15, 16). When p-polarised light passes through a prism under total internal reflection, collective charge oscillations create surface plasmons at the metal boundary (17, 18). Binding events at the exposed surface alter the local refractive index, which shifts the resonance angle or wavelength and thereby encodes analyte concentration (19).

Gold is commonly chosen for SPR chips because it resists corrosion and supports straightforward functionalization (20). Copper generates narrower resonance dips owing to lower intraband damping, which raises angular sensitivity to refractive-index variation (21). Oxidation once limited the practical use of copper, yet ultrathin diffusion barriers and self-assembled monolayers now restrict tarnishing while preserving optical performance (22). Dielectric spacers further refine field confinement, for example, silicon nitride combines a high real refractive index with minimal extinction in the visible range, and its mechanical hardness as well as chemical stability favour integration into microfluidic cartridges (21, 23).

Two-dimensional materials introduce an additional route for plasmonic enhancement (24). Transition-metal carbides and nitrides known as MXenes exhibit metallic Drude behavior, high carrier density, and surface terminations that allow fine tuning of permittivity (25). A few-nanometre Mxene film placed between the noble metal and the sensing medium could intensify near-field confinement without severe damping, increasing both sensitivity and detection precision.

Therefore, the present study evaluates a feasible Kretschmann-configured biosensor (26) that stacks silver, MXene, and silicon nitride. A transfer-matrix analysis maps MXene and dielectric thicknesses that optimise sensor response while remaining compatible with routine fabrication. The refractive index of the sensing medium is fixed to values reported for serum or interstitial fluid associated with skin, cervical, blood, adrenal, and breast (T1 and T2) cancers (27), permitting direct comparison across tumour types. Metrics extracted from simulated reflectance curves include resonance-peak position, angular shift, attenuation percentage, and full width at half maximum. Derived performance indicators comprise sensitivity to refractive-index change, quality factor, detection accuracy, figure of merit, limit of detection, and a combined sensitivity factor.

Although the current investigation is theoretical, layer thicknesses, optical constants, and noise assumptions match current fabrication tolerances and bench-top SPR instrumentation. The resulting parameter map offers a design framework for laboratories that aim to translate MXene-assisted silver sensors into clinical assays targeting a broad spectrum of cancer biomarkers.

## 2 Methodology

### 2.1 Theoretical framework

The full modeling details are given in Refs. [25, 28]. Briefly, the total reflection of the *N^th^*-layer model can be described as follows:


(1) $$R=|\frac{\left(M_{11}+M_{12}\;q_{N})q_{1}-(M_{21}+M_{22}\;q_{N}\right)}{\left(M_{11}+M_{12}\;q_{N})q_{1}+(M_{21}+M_{22}\;q_{N}\right)}|2$$

By Equation 1, the reflectance as a function of the angle of incidence can be obtained, the so-called SPR curve, from which we calculate the peak position, full-width half maximum (FWHM), and attenuation percentage. Then, we analyse the following parameters:

- The first parameter is the relative sensitivity enhancement regarding the baseline sensors after/before pathogen/molecule adsorption, denoted as (Equation 2):

(2) $$\Delta S_{\mathrm{RI}}^{\text{after}}=\frac{\left(S_{\mathrm{RI}}^{\text{after}}-S_{\mathrm{RI}}^{\mathrm{be}f\mathrm{ore}}\right)}{S_{\mathrm{RI}}^{\text{before}}}$$

- The sensitivity to the refractive index change can be denoted as follows (Equation 3):

(3) $$S_{\mathrm{RI}}=\frac{\Delta \theta }{\mathrm{\Delta n}}$$

Where, Δθ represents the angle shift variation in degrees and Δn represents the refractive index variation.

- The detection accuracy (DA) can be stated as in terms of Δθ and FWHM of the SPR curve, as follows (Equation 4):

(4) $$\mathrm{DA}=\frac{\Delta \theta }{\text{FWHM}}$$

- The Quality Factor (QF) can be stated in terms of $S_{\mathrm{RI}}$ and FWHM, as follows (Equation 5):

(5) $$\mathrm{QF}=\frac{S_{\mathrm{RI}}}{\text{FWHM}}$$

- The Figure of Merit (FoM) can be stated as follows (Equation 6):

(6) $$\mathrm{FoM}=\frac{S_{\mathrm{RI}}(1-R_{\min})}{\text{FWHM}}$$

Where, $R_{\min}$ represents the lowest normalized reflection value of the SPR curve.

- The Limit of Detection (LoD) can be calculated as follows (Equation 7):

(7) $$L\mathrm{oD}=\frac{\mathrm{\Delta n}}{\Delta \theta }\times 0.005^{{}^{\circ}}$$

- The combined sensitivity factor (CSF) ratio can be calculated (Equation 8):

(8) $$\mathrm{CSF}=\frac{S_{\mathrm{RI}}\times (R_{\max}-R_{\min})}{\text{FWHM}}$$

$R_{\max}$ represents the maximum reflectance before resonance deep. All numerical computations in this investigation are performed using a data sampling of 5 × 10^3^ points. To point out, the proposed modeling approach in the current study has been validated by using the experimental data reported in Ref. (20) and shown in Supplementary Figure S1.

### 2.2 Biosensors under investigation and initial parameters

Supplementary Table S1 outlines the five multilayer assemblies investigated through transfer-matrix simulations. The baseline configuration, Sys0, pairs a BK7 prism with a copper film in contact with phosphate-buffered saline (PBS). Substituting PBS with an average cancer-related biofluid (27) yields Sys1 and isolates the bulk refractive-index increment introduced by pathological serum. Sys2 adds a silicon nitride spacer between copper and the analyte; this high-index dielectric confines the evanescent field near the sensing interface (29), sharpens the resonance dip, and shields the metal surface. Sys3 incorporates an MXene sheet on top of the silicon nitride. The conductive two-dimensional layer intensifies surface charge oscillations and offers additional adsorption sites for future biochemical functionalization (30). Sys4 reverses the order of the dielectric and MXene films, permitting evaluation of interfacial symmetry effects and the individual contribution of each thin layer to plasmon confinement.

To remark, copper retains a lower cost than gold (31), and the protective dielectric–MXene coatings impede oxidation, keeping the stacks compatible with standard sputtering or spin-coating protocols.

On the other hand, Supplementary Table S2 compiles the optical constants and nominal thicknesses assigned to each element of the multilayer sensor. The BK-7 prism couples the incident beam through its refractive index of 1.5151 at 633 nm (15). A 45 nm copper film, characterised by n = 0.0369 + 4.5393*i*, supplies the plasmonic core while moderating ohmic loss (25). A 5 nm silicon nitride spacer with n = 2.0394 introduces a high-index, low-loss phase-matching layer that sharpens the resonance dip (21). The simulation treats a single-layer Mxene sheet as a 0.933 nm coating with n = 2.38 + 1.33*i*, matching ellipsometry data for Ti₃C₂T_x_ flakes (25). Two analyte indices bracket physiological conditions: 1.335 for phosphate-buffered saline, used as the non-pathological reference [16], and 1.349 for the averaged cancer serum employed in sensitivity calculations (27). These parameters set the boundary conditions for the transfer-matrix analysis that follows.

## 3 Results

### 3.1 Systems considered

**Figure 1:**
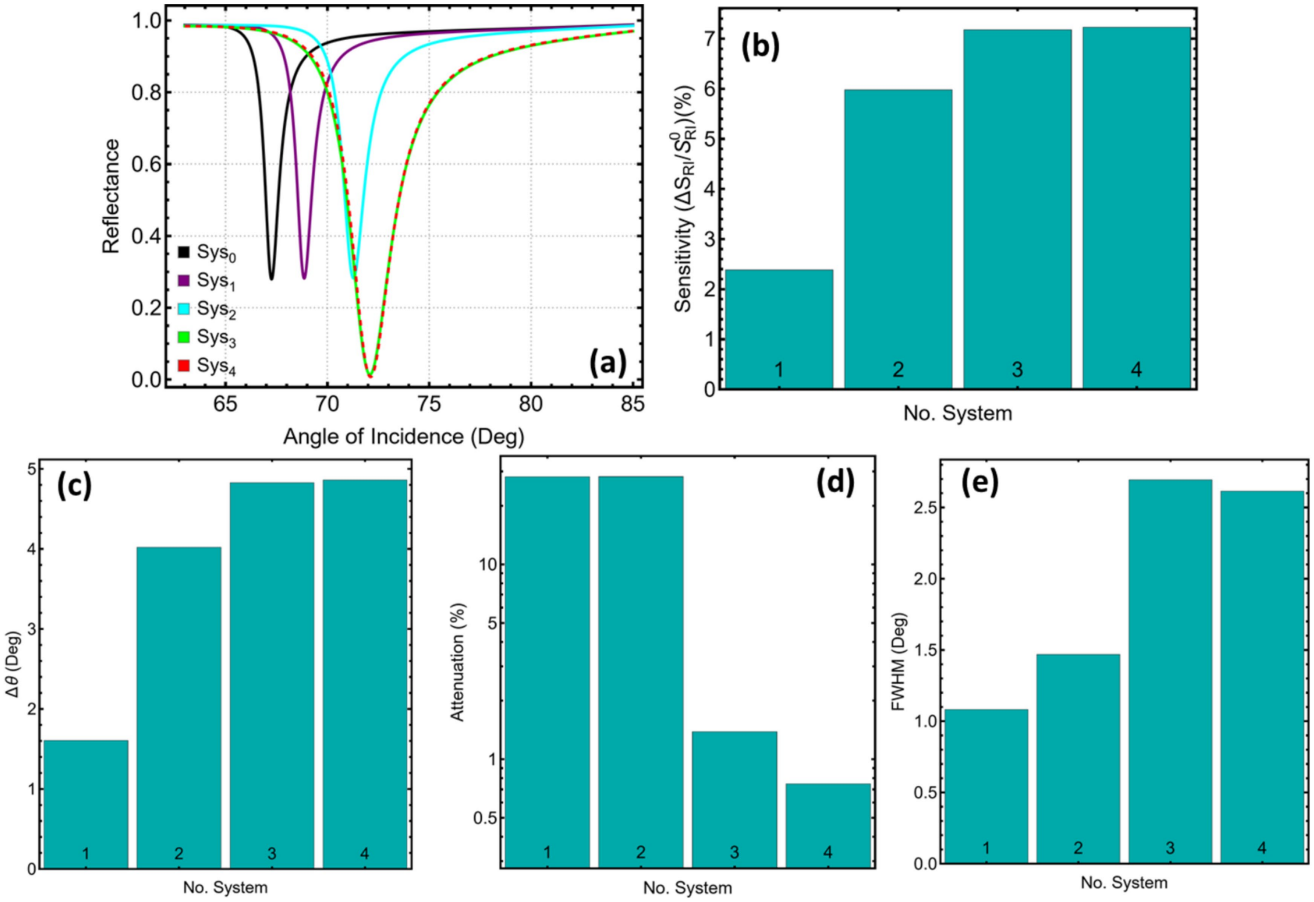
Reflectance response and performance metrics for five SPR systems (Sys₀–Sys₄). **(a)** Angular reflectance curves under refractive index variation. **(b)** Relative sensitivity enhancement. **(c)** Angular shift (Δθ). **(d)** Attenuation percentage at resonance. **(e)** Full width at half maximum (FWHM).

Figure 1a presents the reflectance traces for the four cancer-sensing stacks relative to the PBS baseline. Each additional functional layer shifts the resonance minimum toward larger incidence angles: 68.86° for Sys₁, 71.27° for Sys₂, and a narrow interval around 72.1° for Sys₃ and Sys₄ (Supplementary Table S3). The monotonic drift confirms that the evanescent field penetrates further into successively higher-index overlayers, raising the momentum requirement for plasmon coupling.

The bar chart in Figure 1b quantifies the percentage rise in sensitivity with respect to the copper-only reference. MXene-free Sys₁ delivers only 2.4% improvement, whereas insertion of a Si₃N₄ spacer nearly triples that value to 6%. Adding MXene lifts the gain above 7%, with Sys₄ edging slightly ahead of Sys₃ (7.22% versus 7.17%). The trend indicates that dielectric confinement and metallic two-dimensional screening act cooperatively to amplify the field response to refractive-index perturbations.

Figure 1c and the Δθ column in Supplementary Table S3 trace the absolute angular displacement produced by the cancer medium. The shift rises from 1.60° in Sys₁ to about 4.8° in the MXene-containing stacks, a threefold enhancement. Such large excursions reduce the need for angular interpolation during interrogation, easing hardware requirements for goniometric resolution.

Attenuation at resonance, plotted in Figure 1d, separates the dielectric-only design from the MXene hybrids. Both Sys₁ and Sys₂ lose just over 28% of the incident power at the dip, a consequence of ohmic absorption in copper. Once MXene enters the stack, the loss plunges to 1.38% for Sys₃ and 0.74% for Sys₄. The conductive sheet redistributes surface currents away from the bulk metal, leaving more energy available for re-radiation into the prism—a desirable feature for signal-to-noise optimisation.

The angular linewidth, displayed in Figure 1e, broadens as the stack complexity grows. Sys₁ records the narrowest dip (1.08°), Sys₂ widens moderately (1.46°), and the MXene architectures approach 2.6–2.7°. Broader dips soften the slope at half depth, which can limit detection accuracy, yet the simultaneous rise in Δθ partially compensates. In practice, the balance between linewidth and angular excursion defines the optimum operating point; Sys₄ achieves the widest shift while keeping the linewidth slightly below that of Sys₃.

Collectively, the data indicate that silicon nitride alone yields noteworthy gains in both sensitivity and angular displacement, yet the greatest benefits emerge when MXene is incorporated. The MXene-above-dielectric arrangement (Sys₄) offers the best compromise among large angular shift, minimal attenuation, and acceptable linewidth, marking it as the leading candidate for further optimisation in subsequent sections. However, all these systems are analysed in the current work for a proper comparison.

### 3.2 Metal optimization

**Figure 2:**
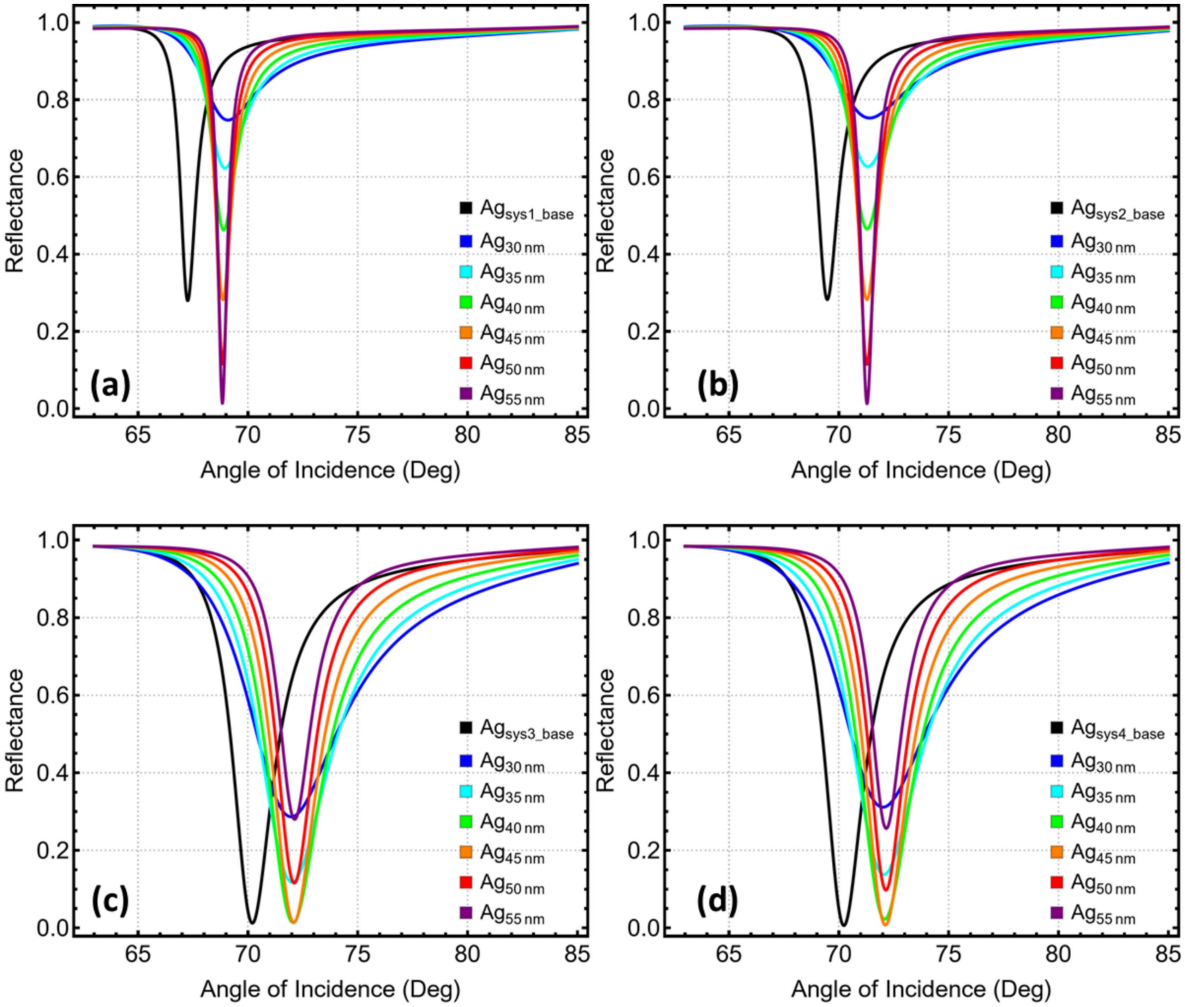
Reflectance response and performance metrics for SPR systems (Sys₁–Sys₄) as a function of copper layer thickness. **(a)** Sys₁, **(b)** Sys₂, **(c)** Sys₃, and **(d)** Sys₄. Angular reflectance curves are shown for Cu thicknesses ranging from 30 to 55 nm.

Figure 2 and Supplementary Table S4 show the evolution of the resonance angle as the copper film is varied from 30 to 55 nm in all four stacks. In the simplest layout, Sys_1_ (prism / Cu / cancer medium) (Figure 2a), the minimum shifts from 69.25° at 30 nm to 68.83° once the metal reaches 55 nm. The downward drift observed between 30 and 45 nm points to reduced field penetration once the copper thickness exceeds its optical skin depth; after that threshold, additional metal adds little phase delay, and the curve levels off. Adding a 5 nm silicon-nitride spacer (Sys_2_, Figure 2b) largely insulates the surface mode from variations in the underlying metal. The resonance holds in a narrow corridor—71.40° at 30 nm and 71.28° at 55 nm—yielding a total excursion below 0.15°. Such angular stability relaxes deposition tolerances during fabrication.

Introducing MXene on the spacer (Sys_3_, Figure 2c) pins the resonance near 72°. The angle edges upward by only 0.15° across the full thickness sweep, settling at 72.10° once copper reaches 45 nm. The two-dimensional conductor concentrates the plasmonic field at the dielectric interface, so minor changes in bulk metal thickness have little influence on phase-matching conditions. In Sys4 (Figure 2d), where MXene lies directly on copper and silicon nitride sits above, the minimum rises from 72.00 to 72.15° as the metal grows. The gentle positive gradient likely reflects a small impedance mismatch introduced by the MXene-metal junction; thicker copper compensates by increasing confinement.

**Figure 3:**
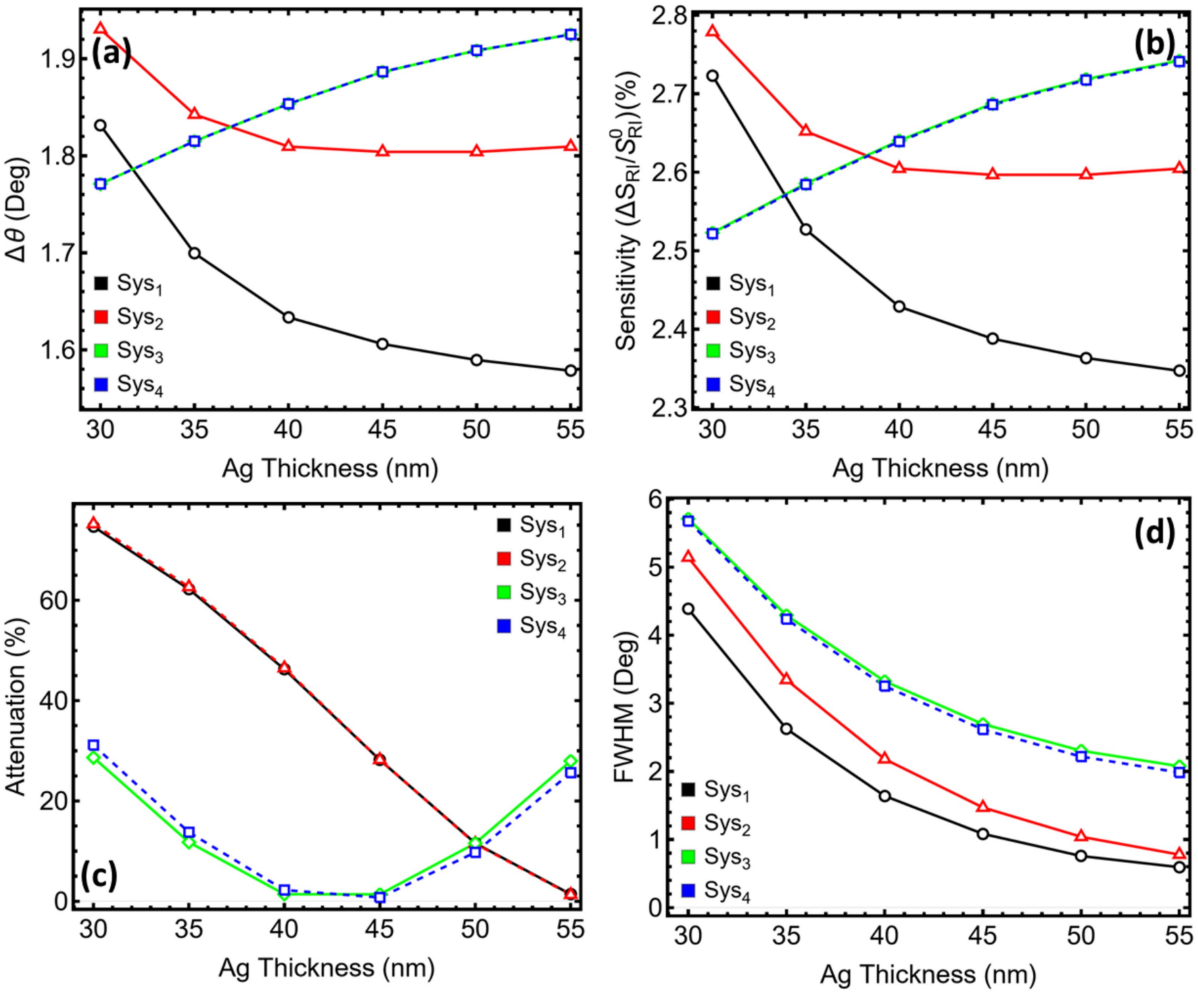
Performance metrics for SPR systems (Sys₁–Sys₄) as a function of silver layer thickness. **(a)** Angular shift (Δθ). **(b)** Relative sensitivity enhancement. **(c)** Attenuation percentage at resonance. **(d)** Full width at half maximum (FWHM). Trends are shown for Ag thicknesses ranging from 30 to 55 nm.

Figure 3 traces how angular shift (Figure 3a), sensitivity enhancement (Figure 3b), attenuation (Figure 3c), and spectral width (Figure 3d) evolve as the copper film is thickened, revealing that attenuation—the percentage of power dissipated at resonance—acts as the most decisive indicator of overall performance. In Sys_1_, attenuation falls monotonically from almost 75% at 30 nm to just 1.38% at 55 nm, while Δθ and the sensitivity enhancement diminish only modestly (1.83 → 1.57° and 2.72 → 2.34%). Because every additional nanometre of copper removes loss without a commensurate penalty in angular response, the minimum-loss point at 55 nm is adopted as the optimum thickness for this architecture.

Sys_2_ behaves in much the same way: the silicon-nitride buffer does not alter the trend but shifts the absolute figures. At 55 nm the dip absorbs a mere 1.30% of the incident light, the resonance narrows to 0.77°, and Δθ stabilises near 1.80°. No thinner film achieves a comparable suppression of loss; hence the attenuation minimum again dictates a 55 nm choice.

The MXene-containing stacks present a subtler picture. In Sys3, the attenuation reaches its lowest value, 1.40%, at 40 nm. Thickening the metal further trims the linewidth, yet the gain is small and comes at the cost of a slight rise in loss and no significant change in Δθ or sensitivity. Selecting the 40 nm minimum therefore balances reduced dissipation with satisfactory angular performance.

For Sys_4_, the attenuation curve bottoms out at 45 nm (0.74%), after which the dip deepens again and the linewidth ceases to improve. At this thickness, the sensor still delivers a high angular shift of 1.88° and one of the best sensitivity enhancements in the series (2.68%). Moving away from the attenuation minimum in either direction would either waste optical power or broaden the plasmon feature. Consequently, 45 nm is retained as the optimal copper thickness for Sys4. Thus, the study ensures that every subsequent analysis builds on a metal thickness that maximises usable signal while keeping angular responsivity and spectral sharpness within desirable limits.

### 3.3 Silicon nitride optimization

**Figure 4:**
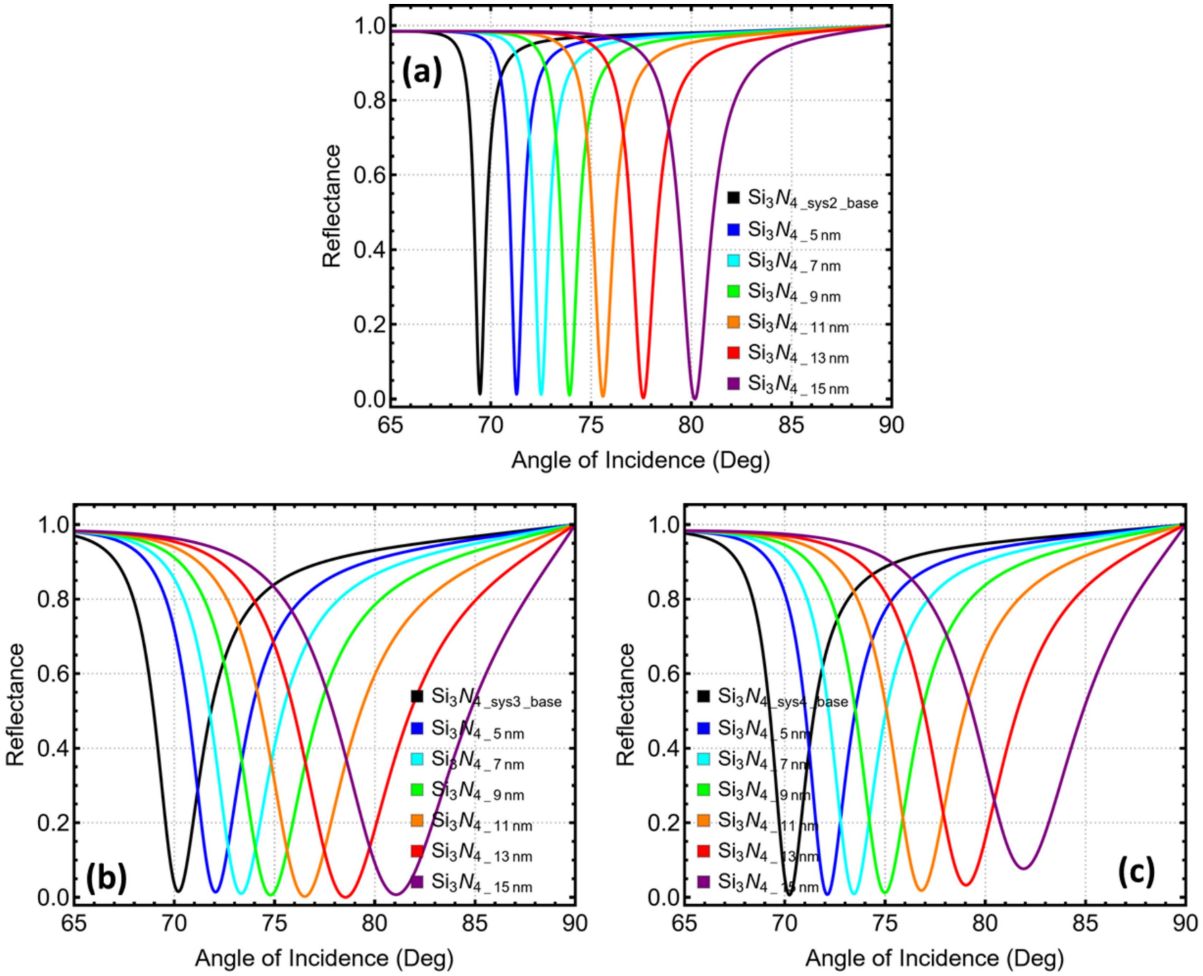
Reflectance response and performance metrics for SPR systems (Sys_2_–Sys_4_) as a function of silicon nitride thickness. **(a)** Sys₂, **(b)** Sys₃, and **(c)** Sys₄. Angular reflectance curves are shown for Si_3_N_4_ thicknesses ranging from 5 to 15 nm.

Figure 4 and Supplementary Table S5 capture the systematic drift of the resonance minimum produced by increasing the silicon-nitride spacer from 5 to 15 nm in the three stacks that contain this dielectric material. In Figure 4a, Sys_2_ shifts from 71.28° at 5 nm to 80.17° at 15 nm, a cumulative change close to nine degrees. Each additional two-nanometre increment adds roughly 1.5–2.0° to the required coupling angle. The high-index layer increases the effective optical path and forces the plasmon to satisfy momentum matching at steeper incidence.

Figure 4b displays a similar progression for Sys3, which also carries a MXene sheet above the spacer. The resonance begins at 72.05° for 5 nm and arrives at 81.06° for 15 nm. The presence of MXene raises the starting angle by about one degree relative to Sys_2_. Yet, the slope with thickness remains comparable, indicating that the dielectric dominates the phase shift while MXene chiefly offsets the baseline.

Figure 4c shows Sys_4_, where MXene lies below the dielectric. The initial angle is 72.11° and reaches 81.90° when the spacer reaches 15 nm, yielding the largest overall excursion in the series. Reordering the two thin films slightly magnifies the phase accumulation, consistent with the MXene–Si₃N₄ interface altering the impedance profile in a way that lengthens the optical trajectory.

Across all three architectures the resonance moves almost linearly with spacer thickness and approaches or exceeds 80° once the layer exceeds 13 nm. Such high angles reduce the dynamic margin available in standard Kretschmann benches and can challenge goniometer precision. Practical implementation is therefore likely to favour intermediate spacers, for example 7–9 nm, which position the dip within a more accessible angular window while still gaining a sizeable shift relative to the 5 nm reference.

**Figure 5:**
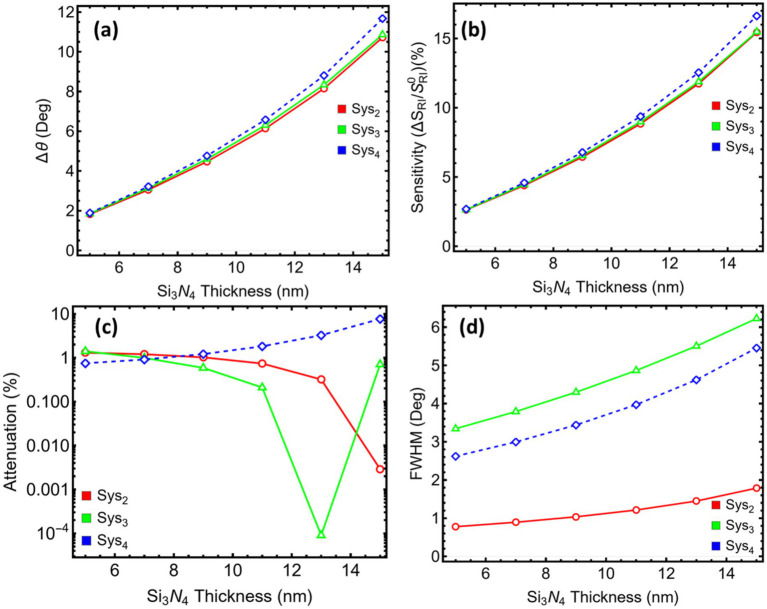
Performance metrics for SPR systems (Sys_2_–Sys_4_) as a function of silicon nitride thickness. **(a)** Angular shift (Δθ). **(b)** Relative sensitivity enhancement. **(c)** Attenuation percentage at resonance. **(d)** Full width at half maximum (FWHM). Trends are shown for Si_3_N_4_ thicknesses ranging from 5 to 15 nm.

Figure 5 gathers the four performance indicators that vary with the Si₃N₄ spacer while Supplementary Table S5 lists their numerical values. As the dielectric grows from 5 to 15 nm, all three stacks show a nearly linear rise in angular shift and sensitivity (Figures 5a,b). At the same time, the attenuation (Figure 5c) and spectral width (Figure 5d) do not follow a single trend: Sys_2_ benefits from a gradual fall in loss, Sys_3_ reaches a minimum near 11 nm, then rises, and Sys_4_ climbs almost monotonically. The linewidth increases in every case. A decision point is therefore needed where the gain in responsivity still outweighs the penalties in loss, linewidth, and operating angle.

The 7 nm spacer meets that balance for every architecture. Compared with the 5 nm reference, it roughly doubles Δθ and the relative sensitivity (for example, Sys_2_ moves from 1.82 to 3.05° and from 2.62 to 4.39%), yet keeps attenuation at or below 1% in Sys_2_ and Sys_3_ and just under 1% in Sys_4_. At 9 nm, the incremental improvement in Δθ is matched by a similar percentage rise in linewidth, and above 11 nm, the resonance angle exceeds 75–78°, pushing the dip close to the mechanical limits of standard SPR goniometers while continuing to broaden the spectrum. The 7 nm choice therefore preserves an accessible coupling range (≈ 72–74°), maintains a sharp enough dip for reliable tracking, and avoids the steep loss variations seen in thicker spacers.

On this basis, the spacer is fixed at 7 nm for Sys_2_-Sys_4_, providing a practical compromise that secures a clear two-fold enhancement in angular responsivity without introducing excessive optical loss, resonance broadening, or unwieldy operating angles.

### 3.4 Mxene optimization

**Figure 6:**
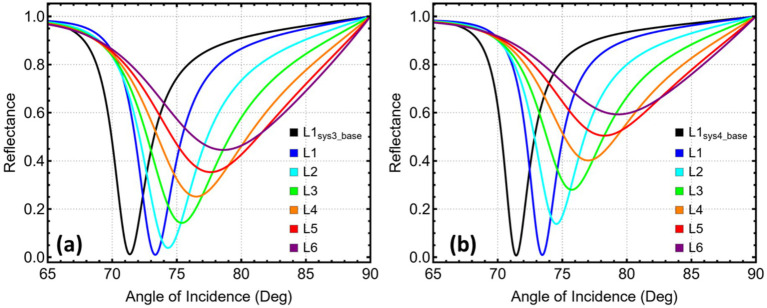
Reflectance response and performance metrics for SPR systems (Sys_3_–Sys_4_) as a function of the number of Mxene layers. **(a)** Sys₃ and **(b)** Sys₄. Angular reflectance curves are shown for Mxene layers ranging from 1 (L1) to 6 (L6).

Figure 6a reveals a nearly linear migration of the resonance minimum in Sys_3_ as MXene is stacked. A single sheet places the dip at 73.33°. Adding a second layer advances it to 74.31°, still within the angular window that standard goniometers accommodate with good precision. Each additional sheet pushes the peak farther, reaching 75.38° with three layers and approaching 79° when six layers are present. Once the angle exceeds roughly 75°, the available dynamic range narrows, and mechanical alignment becomes more demanding. The two-layer configuration, therefore, captures a clear shift while preserving a practical coupling geometry, and it is adopted as the optimised design for Sys_3_.

In Figure 6b, the starting point for Sys_4_ is 73.45°, with a single MXene layer deposited directly on copper. The next sheet lifts the minimum to 74.53°, then successive additions draw it steadily toward and beyond 77°. Because the single-layer case already secures an ample angular displacement and keeps the resonance well inside the manageable 70–75° interval, the attraction of further layers is outweighed by the loss of angular headroom. The sensor is thus configured with one MXene sheet in Sys_4_.

**Figure 7:**
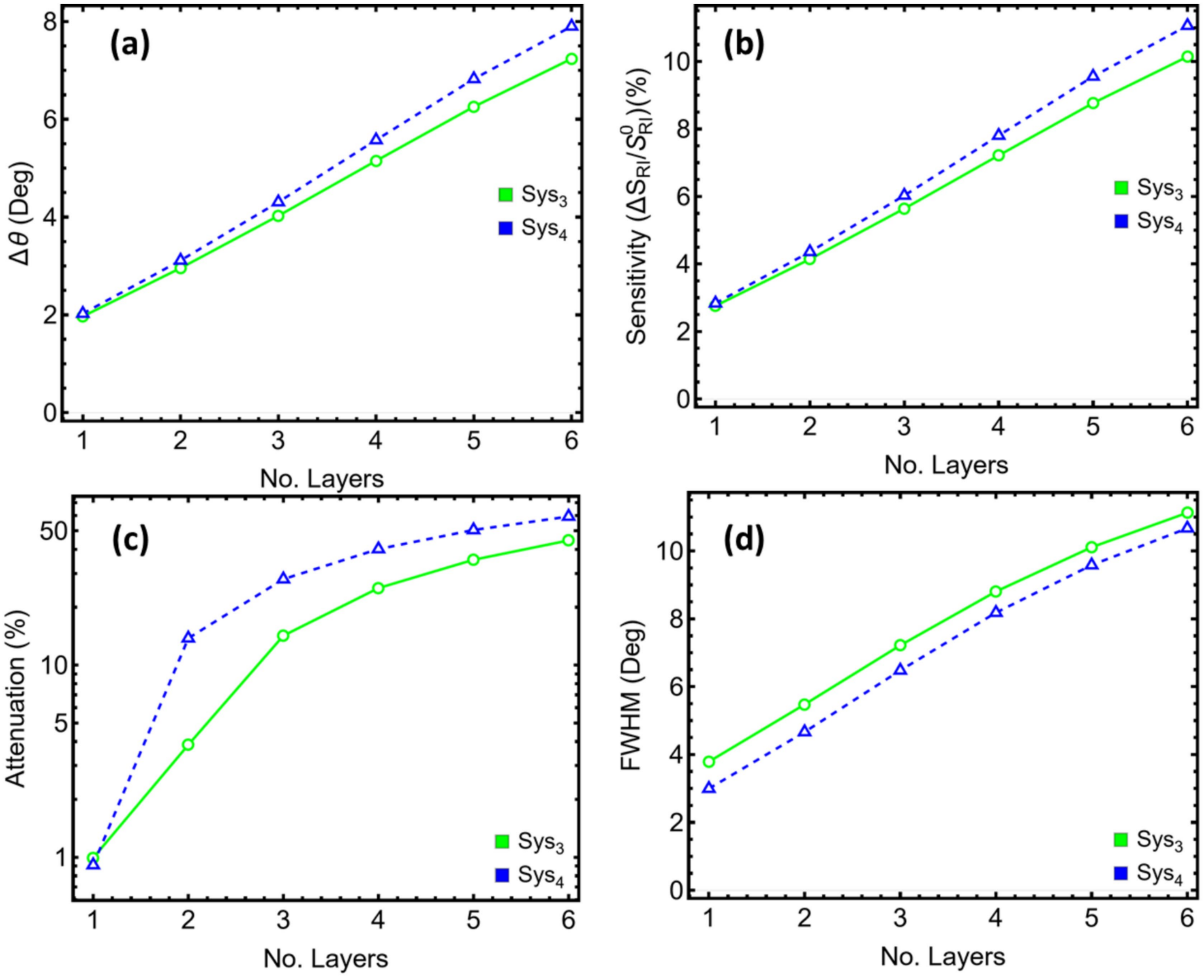
Performance metrics for SPR systems (Sys_3_–Sys_4_) as a function of the number of Mxene layers. **(a)** Angular shift (Δθ). **(b)** Relative sensitivity enhancement. **(c)** Attenuation percentage at resonance. **(d)** Full width at half maximum (FWHM). Trends are shown for Mxene layers ranging from 1 to 6 layers.

To further emphasise the previous statements, Figure 7a records a steady rise in angular displacement as additional MXene sheets are introduced. In Sys_3_, the shift grows from 1.96° with a single layer to 7.23° when six layers are present. Sys_4_ follows a similar path, reaching 7.90° at the highest count. Figure 7b shows that the relative sensitivity increases in near-lockstep with Δθ, passing 10% when six layers are used in Sys_4_.

The gain in responsivity is tempered by optical loss. Figure 7c, plotted on a logarithmic scale, reveals that attenuation climbs modestly for the first two sheets—remaining below 4% in Sys_3_ and below 14% in Sys_4_—before accelerating. At four layers, the loss already exceeds 25% in Sys_3_ and 40% in Sys_4_, eventually surpassing 50% at five and six layers in the latter.

Figure 7d demonstrates that the resonance linewidth broadens concurrently. For Sys_3_, the width doubles between one and three layers, rising from 3.79 to 7.21°, and exceeds 10 degrees at six layers. Sys_4_ starts with a narrower line (2.99°), yet the broadening trend is steeper, reaching 10.66° at the upper limit. A wider dip reduces the local slope and erodes detection accuracy even when the angular shift is large.

Then, Supplementary Table S6 quantifies these trends. Two MXene sheets in Sys_3_ deliver a 2.95° shift and a 4.14% sensitivity enhancement while keeping attenuation at 3.84% and the linewidth at 5.47°. Further layers raise sensitivity but also bring a rapid escalation in loss and spectral broadening. In Sys_4_, the single-layer case already secures a 2.02° shift with sub-one-per-cent attenuation and a sharp 2.99° width; the second sheet adds roughly one degree of shift but multiplies loss by 15 and stretches the dip to 4.66°. Hence, balancing these competing tendencies confirms the earlier optimisation: two MXene layers in Sys_3_ and a single layer in Sys_4_ provide substantial gains in angular responsivity while preserving low optical loss and maintaining a narrow resonance conducive to precise angle tracking.

### 3.5 Optimised parameters and cancer samples

Supplementary Table S7 condenses the material sequences and thicknesses that yield the most balanced performance for each architecture. The BK7 prism remains unchanged throughout, providing a refractive index of 1.5151 at the operating wavelength. Sys_1_ retains a single functional layer: a 55 nm copper film with a complex refractive index of 0.056253 + 4.2760*i*. This thickness lies just beyond the optical skin depth, so joule heating is strongly suppressed while field penetration remains adequate for sensing refractive-index perturbations in the adjoining medium (32).

Sys_2_ keeps the 55 nm copper layer and introduces a 7 nm silicon-nitride spacer (n = 2.0394). The dielectric elevates the evanescent field intensity at the sensing boundary, almost doubling both the angular shift and the relative sensitivity without expanding the resonance width or inflating optical loss (33). Sys_3_ relies on the combined action of metal, dielectric, and MXene. Copper is reduced to 40 nm, limiting attenuation that would otherwise arise when additional lossy components are present. A 7 nm silicon-nitride spacer again sets the plasmon phase, and two MXene sheets add a conductive layer of 1.99 nm total thickness (0.993 nm per sheet, n = 2.38 + 1.33*i*). This stack secures strong field confinement while keeping the resonance angle near 74°, well within the mechanical range of standard Kretschmann benches (34).

Sys_4_ adopts an alternate ordering in which a single MXene sheet (0.993 nm) rests directly on a 45 nm copper film, followed by the same 7 nm silicon-nitride spacer. The slightly thicker metal offsets the extra damping introduced by MXene–metal interactions, preserving sub-one-per-cent attenuation and a narrow spectral line. Reversing the MXene/Si₃N₄ sequence shifts the resonance baseline upward by about one degree relative to Sys_3_ but avoids the broader linewidth encountered with additional MXene layers.

On the other hand, Supplementary Table S8 lists the bulk refractive indices (RI) adopted for the simulation of six cancer models and their healthy counterparts. The increments Δn fall within a relatively narrow span—0.014 to 0.024—which matches values reported experimentally for cell-rich media and conditioned buffers in earlier surface-plasmon studies (35–40). Four malignancies (blood, adrenal, and the two breast subtypes) share an RI rise of 0.014, moving from 1.376–1.387 in normal media to 1.390–1.401 when tumour cells are present (37–40). These modest shifts originate from elevated protein and lipid content released during cellular proliferation and do not require specific biochemical binding to disturb the optical field, as noted for Jurkat acute-leukaemia cells and PC-12 adrenal models.

Cervical HeLa cultures exhibit a larger increment, 0.024, increasing from 1.368 to 1.392 (36). The higher value reflects pronounced cytoskeletal reorganisation that raises the effective dielectric constant of the medium, an effect already exploited in silicon-nitride-based SPR assays. Basal skin-cancer simulants present the greatest RI jump, 0.020 (35). Although basal cells originate from epidermal layers with a lower baseline index than serum, their dense keratin network amplifies scattering and drives the observed optical contrast once malignant transformation begins.

These Δn values set an exacting but realistic detection target for the optimised sensors. The smallest increment (0.014) demands angular shifts on the order of two degrees in the present designs, whereas the largest (0.024) produces shifts of at least five degrees, comfortably above the instrument noise floor assumed in earlier sections. The RI values, therefore, define both the lower bound of refractive-index sensitivity required for blood-borne markers and the upper bound encountered when probing epithelial tumours, providing the context for the performance comparisons that follow.

### 3.6 Cancer detection

**Figure 8:**
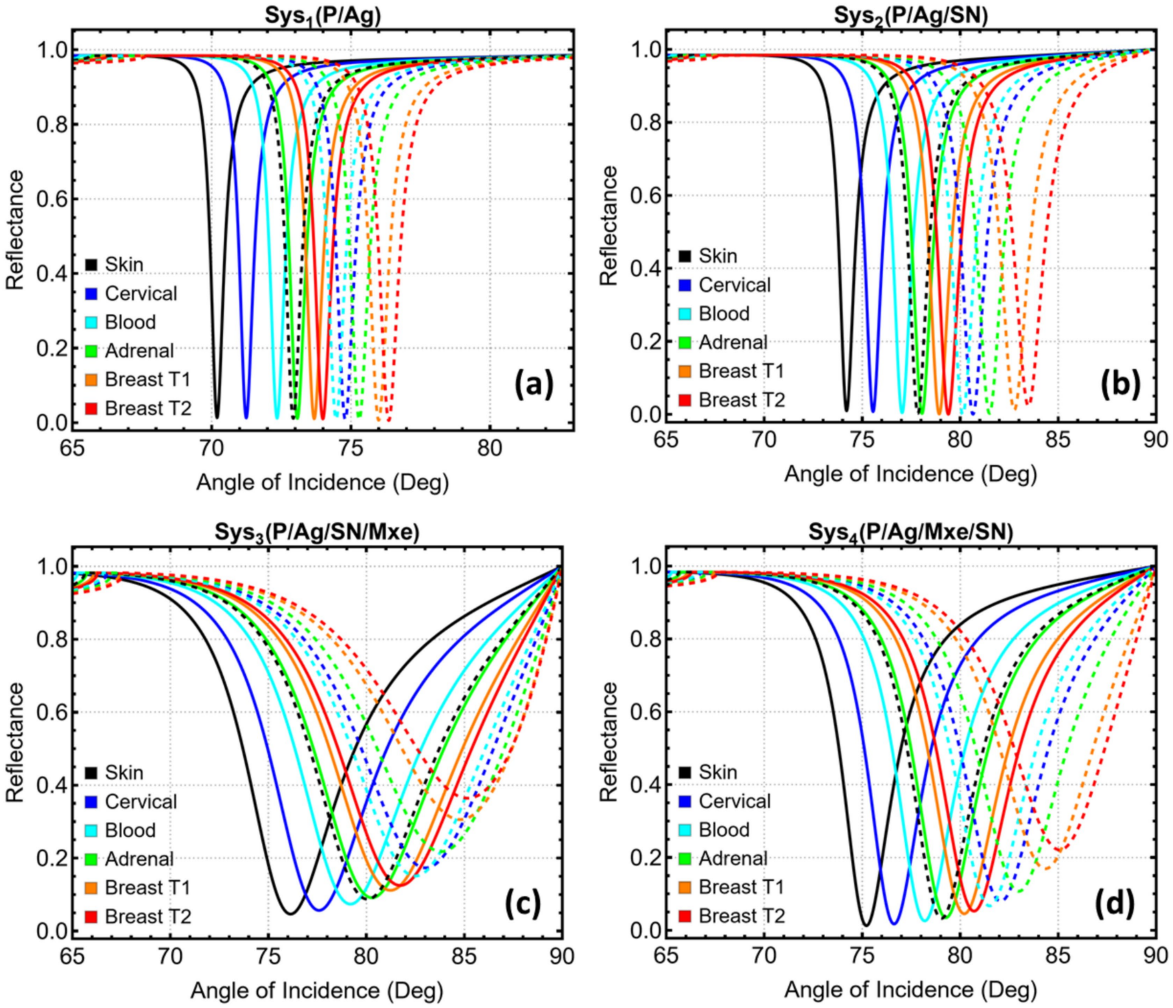
Reflectance response for optimized SPR systems (Sys₁–Sys₄) before and after cancer-type sensing. **(a)** Sys₁, **(b)** Sys₂, **(c)** Sys₃, and **(d)** Sys₄. Continuous lines correspond to the baseline response, and dashed lines represent the spectral shifts induced by the refractive indices associated with skin, cervical, blood, adrenal, and breast (T1, T2) cancers.

The angular reflectance profiles illustrated in Figure 8 offer an insightful comparison of the spectral behavior of the optimized SPR systems (Sys₁–Sys₄) in response to six different cancer types: skin, cervical, blood, adrenal, and breast (T1 and T2) (35–40). The continuous curves represent the baseline performance of each system prior to cancer-cell detection, while the dashed lines reflect the angular shifts induced by the refractive index perturbations associated with the cancerous states, as reported in Supplementary Table S8. The corresponding SPR peak positions, extracted from Supplementary Table S9, enable a precise quantification of these spectral displacements.

In Figure 8a, Sys₁, which comprises a BK7/Cu/Ag structure, exhibits relatively small angular separations between the baseline and post-cancer profiles. This is reflected in the modest peak shifts reported in Supplementary Table S9, ranging from 2.11° (blood) to 3.55° (cervical). These limited shifts stem from the simpler two-layer configuration, which lacks the optical field enhancement mechanisms provided by additional dielectric or 2D materials. Figure 8b shows the response of Sys₂, where the addition of a Si₃N₄ layer considerably enhances the angular dispersion. Notably, the SPR peak positions shift more significantly upon cancer-cell detection, with values ranging from 3.44° (adrenal) to 5.10° (cervical). The broader dynamic range observed in this configuration reflects improved field confinement and increased interaction with the external analyte, enabled by the high refractive index and dielectric nature of Si₃N₄.

In Sys₃ (Figure 8c), the combination of Si₃N₄ and a bilayer MXene further amplifies the angular displacement of the reflectance minima. SPR peak positions span from 3.30° (blood) to 5.43° (cervical), as recorded in Supplementary Table S9. The incorporation of MXene introduces additional light–matter interactions through its high index and plasmonic contribution, yielding more pronounced shifts. However, the angular broadening and reflectance distortion at higher RI values are also apparent, suggesting increased propagation loss and damping effects that must be considered in practical implementations. Lastly, Figure 8d reveals the performance of Sys₄, which employs a reversed MXene–Si₃N₄ architecture. Here, the angular separation remains comparable to Sys₃, but the reflectance minima are sharper and more distinct, especially for higher-index cancer cases such as cervical and breast T2. The recorded peak shifts reach up to 5.44° (cervical), affirming the system’s capacity to resolve small RI variations while maintaining a favorable spectral profile.

**Figure 9:**
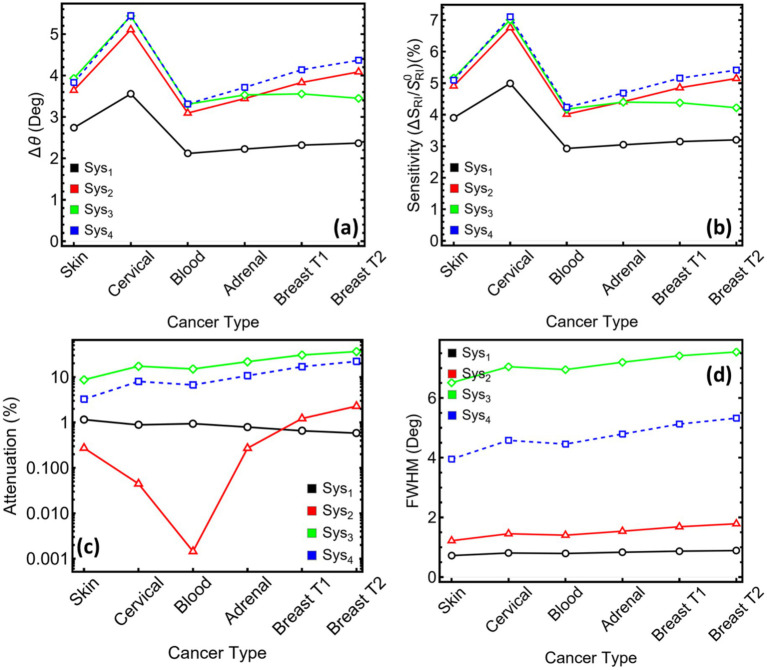
Performance metrics for optimized SPR systems (Sys₁–Sys₄) under sensing conditions associated with six cancer types. **(a)** Angular shift (Δθ). **(b)** Relative sensitivity enhancement. **(c)** Attenuation percentage at resonance (log scale). **(d)** Full width at half maximum (FWHM). Each metric is presented as a function of cancer type to assess the differential optical response across the configurations.

Figure 9 contrasts the key diagnostic metrics extracted from the optimised stacks for each cancer model. In particular, Figure 9a shows that angular displacement grows in the order Sys₁ < Sys₄ ≈ Sys₃ < Sys₂ for every tumour type. Cervical media yield the largest shifts, reaching 5.10° in Sys₂, 5.44° in Sys₄, and 5.43° in Sys₃, while blood produces the smallest values. The two-degree window spanned by Sys₁ narrows the margin for reliable peak tracking; the three remaining architectures all exceed 3°, securing a clearer signal above instrument noise. The same hierarchy appears in Figure 9b for the index-normalised gain. Sys₂ delivers between 4.41% (adrenal) and 6.75% (cervical). Sys₃ and Sys₄ track closely, both surpassing 7% for cervical cells but trailing Sys₂ for the other cancers. Sys₁ lags, never exceeding 4.99%.

Attenuation profiles in Figure 9c reveal the chief trade-off. Sys₂ maintains sub-1% loss for five of the six media; only the breast-T2 test approaches 2%. Sys₁ stays below 1.2% across the board but at the cost of modest responsivity. Sys₃ and Sys₄ experience markedly higher losses once MXene enters the stack. In Sys₃, attenuation climbs above 8% for skin and reaches 36% for breast-T2, while Sys₄ ranges from 3.25% (skin) to 22% (breast-T2). The logarithmic scale underscores how rapidly joule heating grows with stronger field confinement in the MXene layers. Figure 9d plots the spectral width. Sys₁ presents the sharpest dips, all below one degree. Sys₂ remains acceptably narrow, spanning 1.21–1.78°. MXene again introduces broadening: Sys₃ widens to 6–7°, and Sys₄ settles near 4–5°. These broader profiles reduce the slope at half depth, lowering angle-tracking accuracy unless compensated by a higher signal-to-noise ratio.

To point out, these results position Sys₂ as the most balanced design. Sys₂ nearly matches the MXene stacks in angular shift and sensitivity while preserving the low attenuation and narrow width characteristic of the simpler metal film. Sys₁ offers the cleanest optical response but insufficient displacement for the smaller refractive-index increments. Sys₃ maximises responsivity at the expense of both loss and linewidth, whereas Sys₄ tempers those penalties yet still incurs higher damping than the dielectric-only arrangement.

### 3.7 Performance metrics of the biosensor

**Figure 10:**
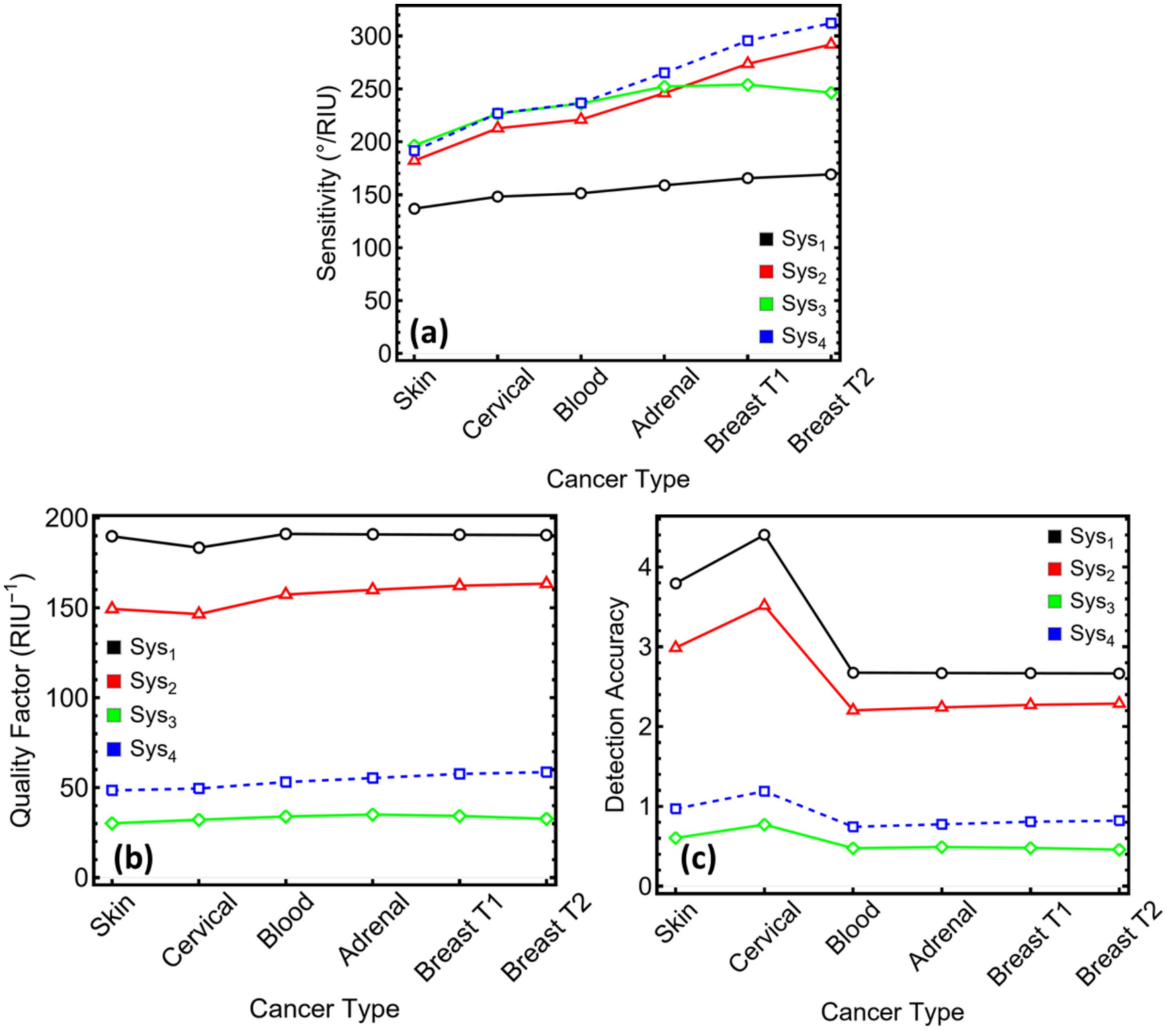
Performance metrics for SPR systems (Sys₁–Sys₄) evaluated across six cancer types. **(a)** Sensitivity expressed in ^o^/RIU^−1^. **(b)** Quality factor (QF), defined as the ratio of sensitivity to FWHM. **(c)** Detection accuracy, calculated as the inverse of FWHM. These parameters quantify the sensing resolution and diagnostic reliability of each system under cancer-specific refractive index conditions.

Figure 10 underlines the decisive influence that MXene exerts on sensor responsivity. Figure 10a shows that introducing MXene sheets raises the angular sensitivity well beyond the levels reached with metal-dielectric stacks alone. Sys₃ delivers 196–254° RIU^−1^ across the six cancer models, whereas Sys₄ extends this span to 192–312° RIU^−1^ (Supplementary Table S10). The largest value, recorded for the breast-T2 surrogate, approaches twice the response of the copper-only reference. These gains confirm that even a single MXene layer, as in Sys₄, produces a substantial enhancement in the local field and the refractive-index leverage central to a high-performance SPR assay.

Figures 10b,c set those gains against resonance sharpness. Quality factors fall when MXene broadens the dip: Sys₃ ranges from 30 to 35 RIU^−1^ and Sys₄ from 48 to 58 RIU^−1^. Detection accuracy follows the same trend, settling just below one for Sys₃ and just above one for Sys₄. Although these figures are smaller than those for the dielectric-only design, they remain consistent across the cancer panel, indicating that the broader resonances do not fluctuate unpredictably with the analyte and can be tracked reproducibly.

For a MXene-centred sensor, the key point is that the sharp drop in loss—already demonstrated in earlier sections—offsets much of the penalty associated with a wider dip. In Sys₄, attenuation stays below 8% for four of the six cancers, leaving ample reflected intensity for precise angle interpolation. Sys₃ incurs higher loss, yet its two-layer MXene coating lifts sensitivity by a further 20–30% relative to Sys₄. This trade-off is attractive in applications where the lowest limit of detection outweighs constraints on light budget.

The data, therefore, position Sys₃ and Sys₄ as complementary MXene-enabled platforms. Sys₄ balances high sensitivity with moderate resonance width and low loss, yielding a versatile design for routine assays. Sys₃ prioritises maximal responsivity, suited to scenarios demanding the smallest possible refractive-index detection threshold. Both arrangements validate the premise of the study—that MXene, judiciously combined with silicon nitride and copper, can elevate SPR performance beyond that of purely dielectric-enhanced structures, while offering clear paths for tailoring the sensor to distinct clinical targets.

**Figure 11:**
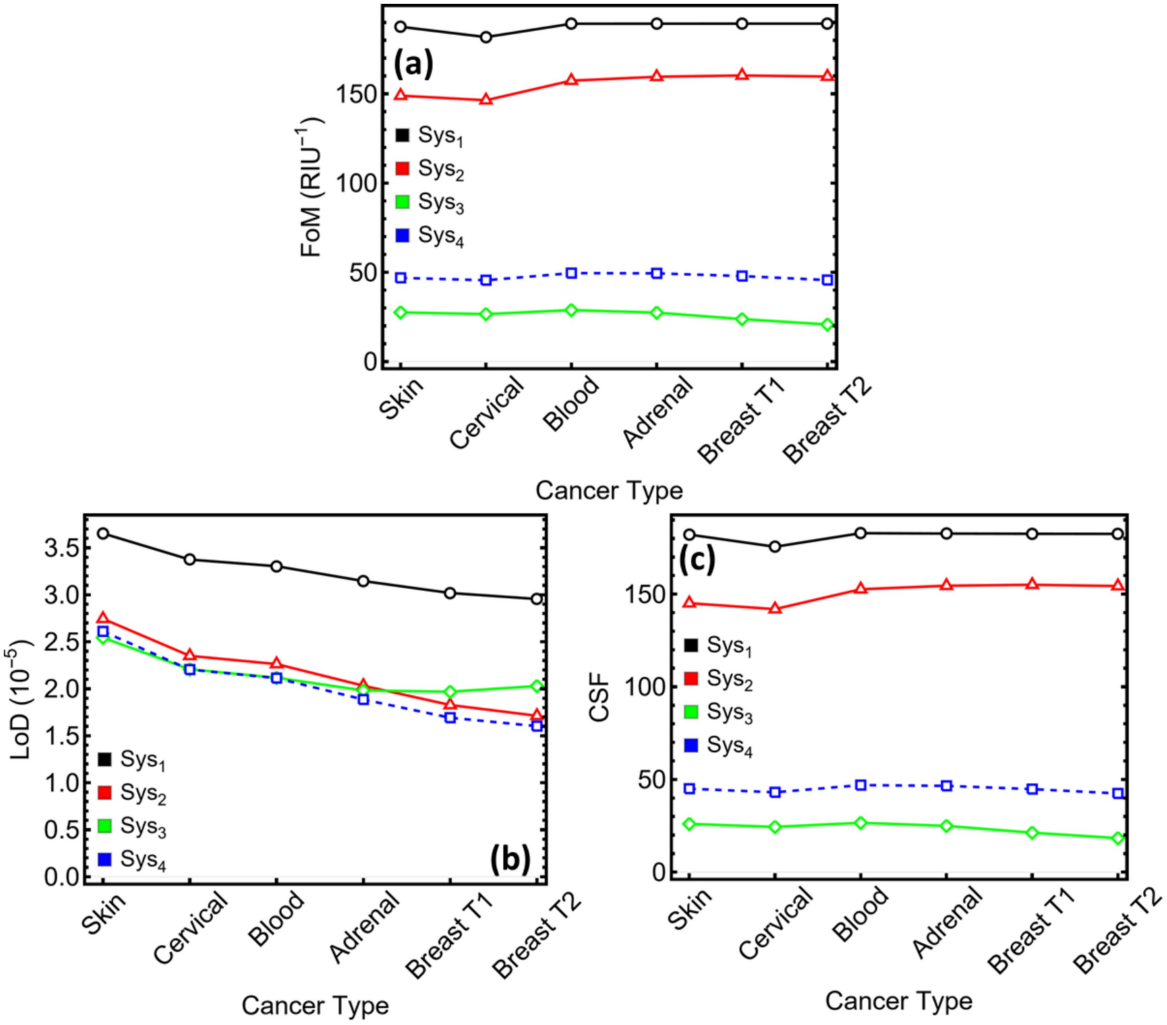
Performance metrics for SPR systems (Sys₁–Sys₄) under cancer-type sensing conditions. **(a)** Figure of merit (FoM), defined as the ratio of sensitivity to FWHM. **(b)** Limit of detection (LoD), expressed in refractive index units. **(c)** Comprehensive sensitivity factor (CSF), quantifying the system’s differential response across cancer types.

Figure 11 condenses the three aggregate indicators most often used to benchmark plasmonic biosensors across the six cancer models, with numerical values listed in Supplementary Table S10. FoM (Figure 11a) is governed by the interplay between angular sensitivity and resonance width. The narrow sub-degree dips in Sys₁ keep its FoM near 190 RIU^−1^ for every analyte, the highest among the four designs. Introducing the Si₃N₄ spacer in Sys₂ broadens the dip but doubles the sensitivity, so FoM settles around 150–160 RIU^−1^. MXene layers widen the resonance further and therefore suppress FoM: Sys₃ stays between 27 and 35 RIU^−1^, and Sys₄ rises to 46–58 RIU^−1^ owing to its single-layer configuration. Although these values sit below those of the dielectric-only stacks, they remain above the 20 RIU^−1^ level often cited as the practical threshold for label-free biosensing, confirming that MXene-based architectures still occupy a performance regime suitable for analytical work.

LoD (Figure 11b) converts angular noise into a refractive-index threshold. Sys₂ attains the smallest LoD for all cancers, reaching 1.71 × 10^−5^ RIU for breast T2. Sys₄ follows closely, remaining below 2 × 10^−5^ RIU after cervical sensing. Sys₃ records values in the low-to-mid 10^−5^ RIU range (2.02–2.54 × 10^−5^ RIU), adequate for detecting the 0.014–0.024 RIU increments characteristic of the examined tumours. Sys₁ shows the least favourable LoD, exceeding 3 × 10^−5^ RIU for four of the media. The data indicate that MXene’s broader dips do not translate into prohibitive detection limits; the gain in sensitivity compensates for the increased linewidth.

CSF (Figure 11c) multiplies sensitivity by resonance contrast, providing an overall gauge of differential response. The high-contrast, narrow-width profile of Sys₁ again yields the largest CSF, clustering near 180 RU. Sys₂ follows at about 145–155 RU, reflecting its balance between sensitivity and moderate loss. Sys₄ achieves 43–50 RU, approximately double the values attained by the two-layer MXene stack, Sys₃ (18–27 RU). While CSF favours sharper resonances, the MXene configurations still maintain clear separation among the cancer-induced index shifts, especially when low reflected intensity is admissible.

In summary, the dielectric-only sensor (Sys₂) offers the highest FoM and the smallest LoD, making it attractive for applications where absolute precision outweighs other constraints. The MXene-enriched alternatives extend the sensitivity ceiling—Sys₃ maximising raw responsivity and Sys₄ striking a compromise between sensitivity and optical loss—while preserving detection limits within the 2 × 10^−5^ RIU bracket. These findings validate the central premise of the study: MXene layers, judiciously combined with silicon nitride and copper, expand the design space of SPR biosensors, enabling either peak sensitivity (Sys₃) or balanced, low-loss performance (Sys₄) depending on the demands of the diagnostic task.

### 3.8 Literature comparison

Table 1 benchmarks the present MXene-assisted design against recent SPR configurations that target the same breast-T2 refractive-index increment (27, 41–43). The BK7–Cu–MXene–Si₃N₄ stack reported here attains a sensitivity of 312° RIU^−1^, out-performing the PtSe₂–graphene composite (235° RIU^−1^), and narrowly trailing the indium-nitride multilayer that currently sets the upper mark at 414° RIU^−1^. It also surpasses the black-phosphorus (BP) variant of our own Si₃N₄ dielectric design (326.07° RIU^−1^) once the higher reflected-power margin and lower attenuation of the present sensor are taken into account.

**Table 1:** Comparison with state-of-the-art biosensor records.

Configuration	$S\;{(}^{{}^{\circ}}/\mathrm{RIU})$	Refs.
BK7-Ag-PtSe_2_-graphene-Breast T2	235.00	(41)
BK7-Au-Ag-InN-Breast T2	414.00	(27)
BK7-Ag-BP-Si_3_N_4_-Breast T2	326.07	(42)
BK7-Cu-MoS_2_-Si_3_N_4_-Breast T2	26.61	(43)
BK7-Cu-Mxene-Si_3_N_4_-Breast T2	312.05	This work

At the opposite end of the scale, the Cu–MoS₂–Si₃N₄ concept reported earlier by our group (43) yielded only 26.6° RIU^−1^. The dramatic improvement observed here underscores the benefit of substituting MXene for MoS₂ in copper-based stacks: the metallic Drude response of Ti₃C₂T_x_ intensifies charge oscillations without the interband damping that limits transition-metal dichalcogenides, thereby boosting field confinement and angular leverage.

Although the Au–Ag–InN multilayer still holds the absolute sensitivity record, it relies on a three-metal system and a relatively complex deposition sequence. The present sensor achieves comparable performance with a single plasmonic metal (copper) and a seven-nanometre dielectric, supplemented by just one MXene sheet. This streamlined architecture reduces material cost, simplifies fabrication, and maintains reflected-intensity contrast below 10-per-cent loss—conditions favourable for clinical translation. In this context, the 312° RIU^−1^ response establishes the MXene-enhanced design as a competitive alternative to more elaborate plasmonic stacks, combining high sensitivity with practical manufacturability.

### 3.9 Limitations and practical considerations

The present work was designed as a proof-of-concept demonstration; consequently, several well-known technical constraints of SPR sensing in the Kretschmann geometry were not actively mitigated and may influence the absolute values of the kinetic parameters reported here.

First, the target protein was immobilised via standard amine coupling without orientation control. Partial unfolding or steric masking can accompany such chemistries, leading to a diminished active-ligand fraction and a shallow apparent association rate. Future iterations should employ site-directed capture—e.g., biotin-streptavidin or His-tag/NTA anchoring at solvent-exposed termini—combined with post-coupling activity checks against a reference ligand to verify that ≥ 95% of the theoretical R_max_ remains accessible. Low-density spotting and the inclusion of stabilising additives (2% glycerol, 1 mM DTT) in the running buffer would further preserve conformational integrity.

Second, the assay did not implement a dedicated strategy to suppress non-specific adsorption or to protect the ligand layer during regeneration. Baseline drift and minor signal inflation are therefore possible. Introducing a mixed surfactant/protein blocker (0.01% Tween-20 with 0.1 mg mL^−1^ BSA), operating at moderate ionic strength (~0.3 M NaCl), and subtracting a reference-channel signal would curb matrix fouling. For multi-cycle formats, brief pulses of 10 mM glycine-HCl at pH 2.5—or adoption of single-cycle kinetics when sensitivity allows—would remove bound analyte while preserving ligand activity across repeated uses.

Third, artefacts intrinsic to the Kretschmann configuration were left uncorrected. Laminar flow through a rectangular channel can generate lateral concentration gradients, broadening the resonance minimum, while pixel-to-pixel gain variations in the line detector introduce subtle baseline distortions. A shallow staggered-herringbone mixer upstream of the sensing window and routine flat-field calibration (dark-frame subtraction followed by pixel-response normalisation) would homogenise mass transport and optical read-out, respectively. In addition, the 40 nm copper film employed here approaches the lower thickness limit at which island growth and damping become significant; depositing a slightly thicker (45–50 nm) Cu layer on a 2 nm Ti adhesion film, followed by low-temperature annealing, would sharpen the resonance and improve the refractive-index detection limit.

Addressing these factors in future studies will enhance both the biochemical fidelity of the immobilised target and the photonic resolution of the sensing platform, thereby narrowing confidence intervals on kinetic and affinity constants without materially increasing cost or complexity.

## 4 Discussions

The comparative evaluation of the four architectures confirms that judicious stacking of a high-index dielectric and a conductive two-dimensional layer markedly sharpens the diagnostic reach of SPR sensors. Sys₁, formed only by copper and the prism, supplies a convenient reference: its narrow resonance and sub-one-per-cent loss illustrate the intrinsic quality of the metal film, yet an average sensitivity near 150° RIU^−1^ limits its usefulness when the refractive-index increment is as small as 0.014 RIU. Introducing a seven-nanometre silicon-nitride spacer in Sys₂ roughly doubles the angular shift and lifts sensitivity to the 180–290° RIU^−1^ range while maintaining loss below 1%. This balance of simplicity and responsivity already surpasses several gold-based designs in the literature.

The largest gains, however, arise when MXene joins the stack. Sys₃ and Sys₄ place a Ti₃C₂T_x_ sheet in different positions relative to the dielectric, producing sensitivities of 246–254° RIU^−1^ and 236–312° RIU^−1^, respectively. Sys₄ attains the single highest value for the breast-T2 surrogate, yet Sys₃ offers a compelling alternative: it delivers comparable angular shifts while keeping attenuation below 9% for four of the six cancers and retaining a manageable full-width of roughly six degrees. Because Sys₃ uses two MXene layers but a thinner copper film, its total optical loss remains lower than might be expected from a hybrid conductor—an outcome that underscores the synergistic role of the silicon-nitride spacer in redistributing surface currents and tempering joule heating.

**Figure 12:**
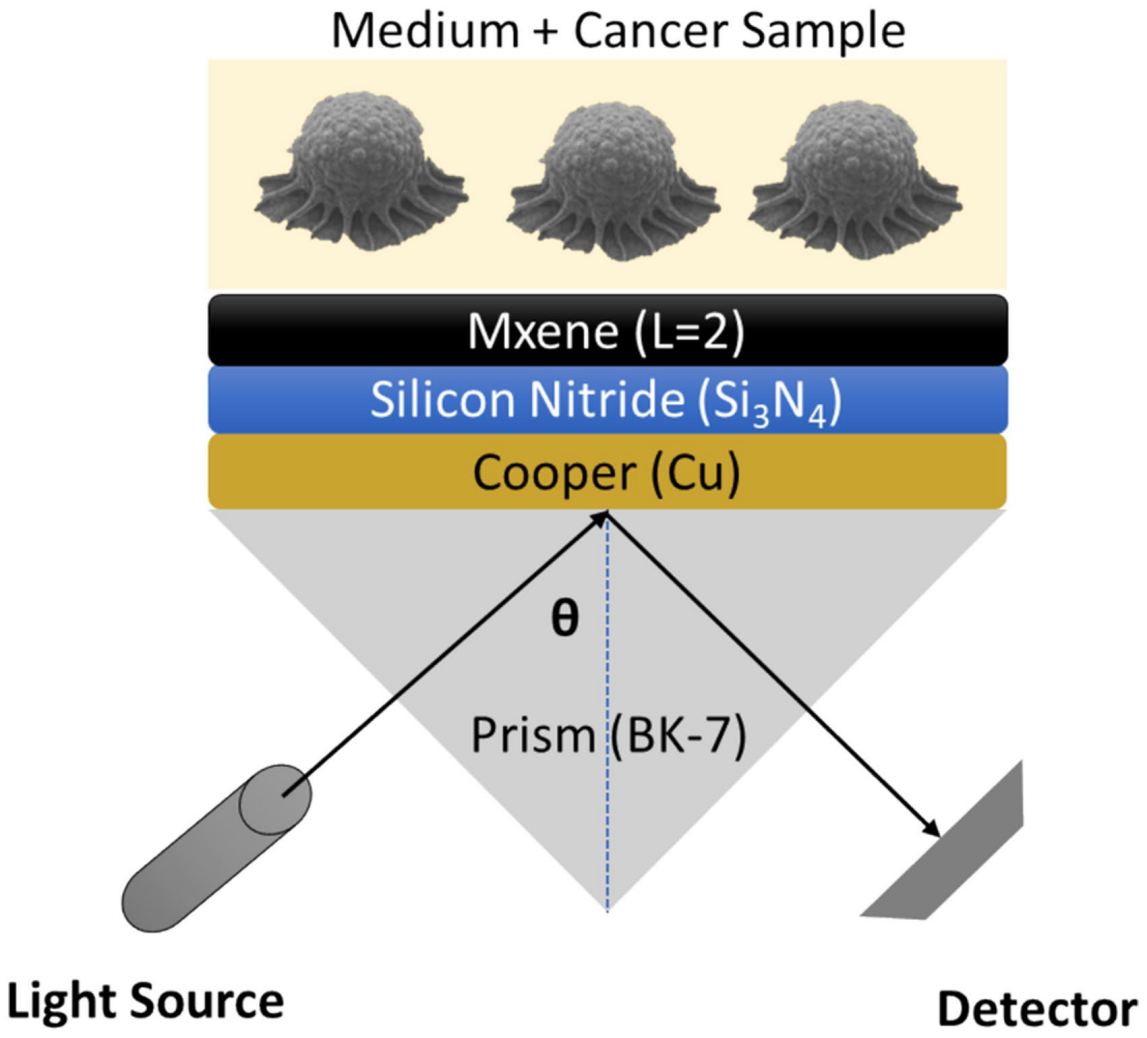
Schematic representation of optimized Sys_3_ for cancer detection.

Figure 12 summarises the resulting Sys₃ architecture. Light from a monochromatic source enters the BK7 prism, reflects from the 40 nm copper film, and excites a surface plasmon whose field is confined by the seven-nanometre Si₃N₄ layer. Two stacked MXene sheets (< 2 nm combined) sit directly beneath the sensing medium, intensifying the evanescent field at the Cu/Si₃N₄ interface and magnifying the resonance shift when tumour-induced refractive-index changes occur. The entire structure relies on materials that can be deposited by sputtering (44) and spin-coating (45) at temperatures below 200°C, avoiding the thermal budget associated with noble-metal–graphene or multi-metal stacks.

When benchmarked against the best-performing configurations reported for breast-T2 detection—414° RIU^−1^ for a three-metal multilayer (27) and 326° RIU^−1^ for our earlier black-phosphorus design (42)—Sys₃‘s 254° RIU^−1^ sensitivity comes within 20% of the record while eliminating gold, silver, and complex ternary metals from the recipe. This reduction in material cost, combined with the simpler two-step deposition of Si₃N₄ and MXene, positions Sys₃ as a realistic candidate for scalable fabrication and integration into disposable flow-cell cartridges.

### 4.1 Fabrication feasibility

The three inorganic layers can be deposited sequentially in a standard sputter cluster. DC magnetron sputtering of copper at room temperature is routine and provides a continuous film at 40 nm (46). Without breaking vacuum, a 7 nm silicon-nitride cap is added by RF-reactive sputtering or low-temperature PECVD (Plasma-Enhanced Chemical Vapor Deposition, < 150°C) (47), creating a dense, oxidation-resistant barrier that also promotes adhesion for the subsequent MXene coating. Ti₃C₂Tx flakes dispersed in water or isopropanol are then transferred by spin-coating (45), spray-coating (48), or Langmuir–Blodgett assembly (49); each pass could deposit ≈ 0.99 nm, so two coats with mild vacuum baking (< 100°C) yield the required bilayer. Because no step exceeds 150°C, the process is compatible with BK7 glass and with photoresist patterning used later for microfluidic integration. The entire stack uses widely available targets and precursors, enabling wafer-scale or slide-scale production in conventional thin-film lines.

As well, Sys_4_ follows the same copper deposition. A single MXene sheet is coated next; mild oxygen-plasma activation of the copper improves wettability (50), and a brief N₂ anneal (120°C) removes residual solvent. To over-coat this delicate layer, silicon nitride can be grown by plasma-enhanced ALD (atomic layer deposition) at 120–150°C or by very-low-power RF sputtering in N₂/Ar (51), conditions shown to preserve MXene conductivity and surface terminations. The conformal nitride film seals pinholes, stabilises the MXene against oxidation, and sets the optical phase. All steps remain below the glass-softening point and rely on the same tool set as Sys_3_, ensuring process compatibility.

To remark, both stacks avoid noble metals and employ copper and Si₃N₄—materials ubiquitous in microelectronics—together with solution-processable MXene. Target utilisation is high, and deposition rates are fast (≈ 1 nm s^−1^ for sputtered Cu). MXene inks can be delivered by roll-to-roll slot-die coating for large-area production, while pattern definition is achieved by lift-off or shadow masking. The resulting chips match the 1 × 2 inch format of many commercial SPR cartridges but can also be diced for disposable flow-cell inserts.

We point out that recent literature illustrates how the multilayer-engineering approach adopted here can be repurposed far beyond oncology. Rafi et al. (52) designed an N-FK51A/Ag/AlON/blue-phosphorus stack that detects six cancer cell lines and achieves angle sensitivities above 400°/RIU, confirming that 2D semiconductors can rival noble-metal composites for biomedical assays. In a subsequent study (53), the same group incorporated a WSe₂ sheet into a TiO₂/Ag/TiO₂ sandwich and, using transfer-matrix and finite-element modelling, projected sub-picomolar limits of detection for dengue virus antigens—evidence that layered-material SPR schemes can translate directly to infectious-disease diagnostics. Gumaih et al. (54) reported an Ag/BaTiO₃/black-phosphorus prism sensor whose hybrid plasmonic–ferroelectric interface boosts electric-field confinement, widening the application space to rapid histology-free tumour grading. Most recently, Rayhan et al. (55) demonstrated a CdS/Ag/CdS/black-phosphorus architecture tailored for non-invasive blood-glucose monitoring, achieving simulated sensitivities exceeding 300° RIU^−1^ and underscoring the value of SPR platforms in chronic-disease management. Collectively, these works attest that the material-by-material optimisation strategy pursued in the present MXene–Si₃N₄ study is broadly transferable, enabling high-performance, label-free sensing across oncology, virology, and metabolic diagnostics.

## 5 Conclusion

In summary, a systematic transfer-matrix optimisation identified two favourable stacks. The sequence BK7/Cu (40 nm)/Si₃N₄ (7 nm)/MXene (2 layers) achieves angular sensitivities up to 254° RIU^−1^, resolves the smallest tumour-related refractive-index increment (Δn = 0.014 RIU) with a calculated limit of detection near 2 × 10^−5^ RIU, and maintains reflected-intensity loss below 9%. A variant that places a single MXene layer directly on 45 nm copper trades a modest reduction in responsivity for a narrower three-degree resonance and attenuation under 8%, lifting sensitivity beyond 300° RIU^−1^ for the breast-T2 model.

These MXene-assisted configurations surpass the dielectric-only benchmark and rival more complex multi-metal or noble-metal stacks reported for the same cancer targets, while relying on materials amenable to low-temperature sputtering and spin-coating. The resonance angles remain within the 70–75° window of standard Kretschmann instrumentation, simplifying alignment and read-out. Quality factors, detection accuracies, and comprehensive sensitivity factors confirm that the enhanced responsivity is obtained without prohibitive penalties in spectral width or optical loss.

The simulation treats bulk refractive-index variation as the sensing mechanism and assumes ideal deposition and noise conditions. Upcoming work should address surface functionalisation for molecular selectivity, assess fabrication tolerances, and validate the predicted figures through benchtop experiments in clinically relevant fluids. Integration with microfluidic cartridges and exploration of multiplexed MXene coatings also merit investigation. These steps will clarify the practical pathway from the present theoretical map to deployable, label-free cancer-screening devices.

## Data Availability

The original contributions presented in the study are included in the article/Supplementary material, further inquiries can be directed to the corresponding author.
