# Supplementary material for: A novel Mxene-SPR-based sensor for sensing different types of cancers
Source: Front Med (Lausanne). 2025 Aug 6;12:1608424. doi: 10.3389/fmed.2025.1608424 (PMC12364876; doi:10.3389/fmed.2025.1608424)
Supplement: Supplementary file 1 [file Supplementary_file_1.docx]

**SUPPLEMENTARY INFORMATION:** **A Novel Mxene-SPR-Based Sensor for Sensing Different Types of Cancers**

Supplementary Figures


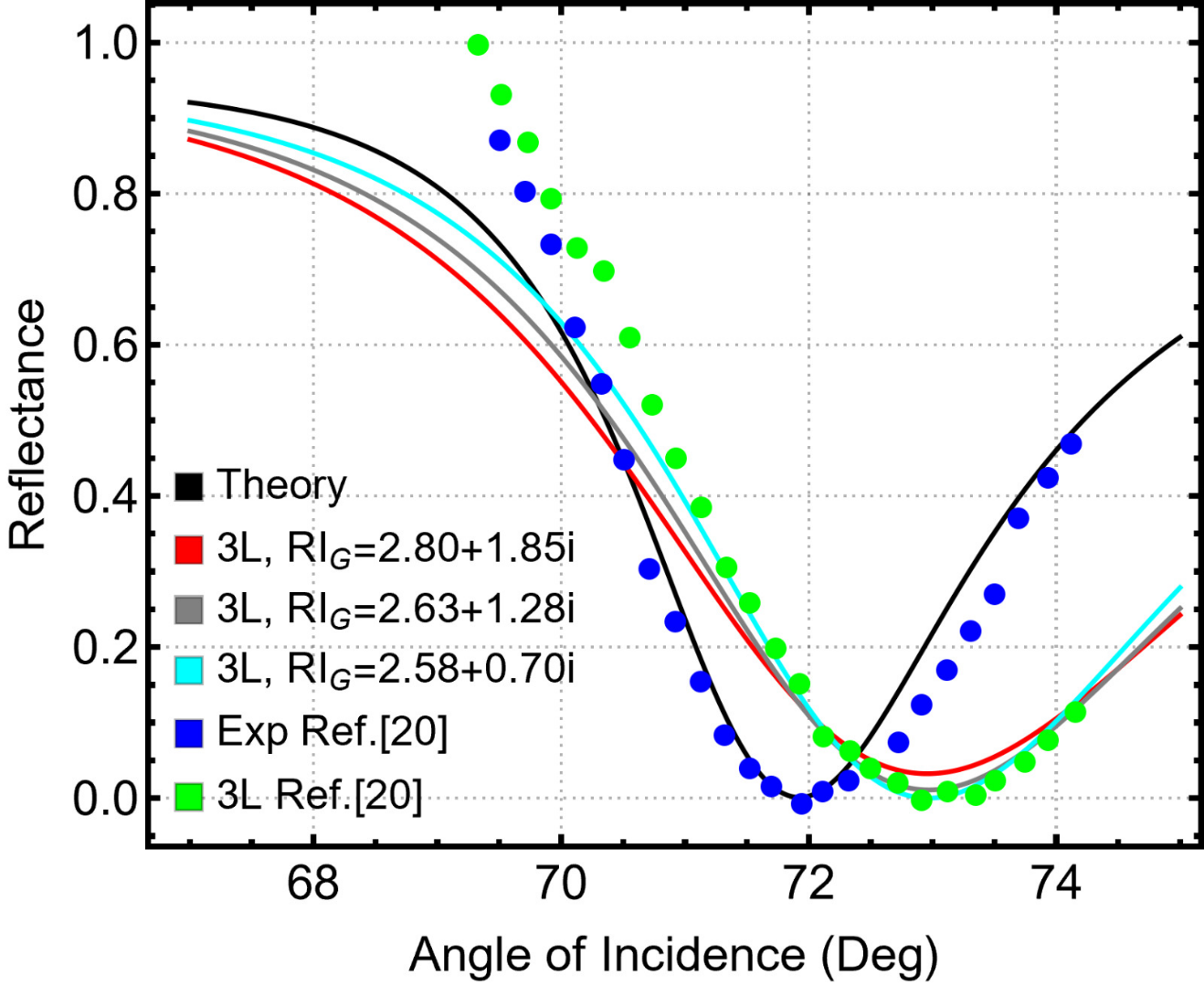


**Figure S1**. The proposed modeling approach was evaluated by using the experimental results from Ref. [20] (see main text).

**Supplementary Tables**

**Table S1.** Systems under study

| **Sys No.** | **Code** | **Full Name** | **Nick Name** |
| --- | --- | --- | --- |
| 0 | Sys_0_ | Prism/Cooper/PBS Medium | P/Cu/M_PBS_ |
| 1 | Sys_1_ | Prism/Cooper/Cancer Sample | P/Cu/M_Cancer_ |
| 2 | Sys_2_ | Prism/Cooper/S_3_N_4_/ Cancer Sample | P/Cu/SN/M_Cancer_ |
| 3 | Sys_3_ | Prism/Cooper/S_3_N_4_/Mxene/Cancer Sample | P/Cu/SN/Mxene/M_Cancer_ |
| 2 | Sys_4_ | Prism/Cooper/Mxene/S_3_N_4_/Cancer Sample | P/Cu/Mxene/SN/M_Cancer_ |

**Table S2.** Initial parameters of the main components of the SPR Biosensor

| **Material** | **Refractive Index** | **Thickness (nm)** | **Ref.** |
| --- | --- | --- | --- |
| BK-7 (P) | 1.5151 | --- | [15] |
| Cooper (Cu) | 0.0369 + 4.5393i | 45.0 | [25] |
| Si_3_N_4_ (SN) | 2.0394 | 5.00 | [21] |
| Mxene | 2.38 + 1.33 i | 0.933 | [25] |
| PBS (M) | 1.335 | --- | [16] |
| Cancer Sample ($\Delta n$) | 1.349 | --- | [27] |

**Table S3.** Metrics of the different systems under consideration

| **Sys No.** | **Code** | **SPR Peak position** | **Sensitivity Enhancement (%)** | $\boldsymbol{\Delta\theta}$ **(Deg)** | **Attenuation (%)** | **FWHM** |
| --- | --- | --- | --- | --- | --- | --- |
| 1 | Sys_1_ | 68.86 | 2.38 | 1.60 | 28.18 | 1.08 |
| 2 | Sys_2_ | 71.27 | 5.97 | 4.02 | 28.21 | 1.46 |
| 3 | Sys_3_ | 72.08 | 7.17 | 4.82 | 1.38 | 2.69 |
| 4 | Sys_4_ | 72.11 | 7.22 | 4.86 | 0.74 | 2.61 |

**Table S4.** Metrics of the different systems under consideration by varying the cooper thickness

| **Sys No.** | **SPR Peak position** | **Sensitivity Enhancement (%)** | $\boldsymbol{\Delta\theta}$ **(Deg)** | **Attenuation (%)** | **FWHM** |
| --- | --- | --- | --- | --- | --- |
| **Sys_1_** | | | | | |
| 30 | 69.25 | 2.72 | 1.83 | 74.71 | 4.38 |
| 35 | 68.95 | 2.52 | 1.69 | 62.25 | 2.62 |
| 40 | 68.89 | 2.42 | 1.63 | 46.26 | 1.63 |
| 45 | 68.86 | 2.38 | 1.60 | 28.18 | 1.08 |
| 50 | 68.84 | 2.36 | 1.58 | 11.57 | 0.75 |
| 55 | 68.83 | 2.34 | 1.57 | 1.38 | 0.59 |
| **Sys_2_** | | | | | |
| 30 | 71.40 | 2.77 | 1.93 | 75.23 | 5.14 |
| 35 | 71.31 | 2.65 | 1.84 | 62.67 | 3.34 |
| 40 | 71.28 | 2.60 | 1.80 | 46.50 | 2.17 |
| 45 | 71.27 | 2.59 | 1.80 | 28.21 | 1.46 |
| 50 | 71.27 | 2.59 | 1.80 | 11.46 | 1.03 |
| 55 | 71.28 | 2.60 |  | 1.30 | 0.77 |
| **Sys_3_** | | | | | |
| 30 | 71.97 | 2.52 | 1.77 | 28.65 | 5.70 |
| 35 | 72.01 | 2.58 | 1.81 | 11.77 | 4.29 |
| 40 | 72.05 | 2.64 | 1.85 | 1.40 | 3.32 |
| 45 | 72.08 | 2.68 | 1.88 | 1.38 | 2.69 |
| 50 | 72.10 | 2.71 | 1.90 | 11.56 | 2.30 |
| 55 | 72.12 | 2.74 | 1.92 | 27.98 | 2.07 |
| **Sys_4_** | | | | | |
| 30 | 72.00 | 2.52 | 1.77 | 31.12 | 5.67 |
| 35 | 72.04 | 2.58 | 1.81 | 13.75 | 4.23 |
| 40 | 72.08 | 2.63 | 1.85 | 2.23 | 3.25 |
| 45 | 72.11 | 2.68 | 1.88 | 0.74 | 2.61 |
| 50 | 72.14 | 2.71 | 1.90 | 9.74 | 2.21 |
| 55 | 72.15 | 2.74 | 1.92 | 25.63 | 1.98 |

**Table S5.** Metrics of the different systems under consideration by varying the silicon nitride thickness

| **Sys No.** | **SPR Peak position** | **Sensitivity Enhancement (%)** | $\boldsymbol{\Delta\theta}$ **(Deg)** | **Attenuation (%)** | **FWHM** |
| --- | --- | --- | --- | --- | --- |
| **Sys_2_** | | | | | |
| 5 | 71.28 | 2.62 | 1.82 | 1.30 | 0.77 |
| 7 | 72.50 | 4.39 | 3.05 | 1.20 | 0.89 |
| 9 | 73.92 | 6.43 | 4.46 | 1.02 | 1.03 |
| 11 | 75.59 | 8.83 | 6.13 | 0.73 | 1.21 |
| 13 | 77.60 | 11.73 | 8.15 | 0.32 | 1.44 |
| 15 | 80.17 | 15.43 | 10.71 | 0.002 | 1.78 |
| **Sys_3_** | | | | | |
| 5 | 72.05 | 2.64 | 1.85 | 1.40 | 3.34 |
| 7 | 73.33 | 4.46 | 3.12 | 0.99 | 3.79 |
| 9 | 74.80 | 6.55 | 4.60 | 0.58 | 4.29 |
| 11 | 76.50 | 8.98 | 6.30 | 0.21 | 4.86 |
| 13 | 78.53 | 11.87 | 8.33 | 0.00 | 5.50 |
| 15 | 81.06 | 15.47 | 10.86 | 0.70 | 6.22 |
| **Sys_4_** | | | | | |
| 5 | 72.11 | 2.68 | 1.88 | 0.74 | 2.62 |
| 7 | 73.45 | 4.58 | 3.21 | 0.91 | 2.99 |
| 9 | 74.99 | 6.78 | 4.76 | 1.21 | 3.43 |
| 11 | 76.81 | 9.37 | 6.58 | 1.82 | 3.97 |
| 13 | 79.03 | 12.53 | 8.80 | 3.25 | 4.62 |
| 15 | 81.90 | 16.62 | 11.67 | 7.65 | 5.45 |

**Table S6.** Metrics of the different systems under consideration by varying the number of Mxene layers

| **Sys No.** | **SPR Peak position** | **Sensitivity Enhancement (%)** | $\boldsymbol{\Delta\theta}$ **(Deg)** | **Attenuation (%)** | **FWHM** |
| --- | --- | --- | --- | --- | --- |
| **Sys_3_** | | | | | |
| L1 | 73.33 | 2.75 | 1.96 | 0.99 | 3.79 |
| L2 | 74.31 | 4.14 | 2.95 | 3.84 | 5.47 |
| L3 | 75.38 | 5.64 | 4.02 | 14.22 | 7.21 |
| L4 | 76.51 | 7.21 | 5.15 | 25.16 | 8.80 |
| L5 | 77.61 | 8.76 | 6.25 | 35.33 | 10.10 |
| L6 | 78.60 | 10.14 | 7.23 | 44.55 | 11.12 |
| **Sys_4_** | | | | | |
| L1 | 73.45 | 2.83 | 2.02 | 0.91 | 2.99 |
| L2 | 74.53 | 4.35 | 3.11 | 13.79 | 4.66 |
| L3 | 75.73 | 6.02 | 4.30 | 28.01 | 6.47 |
| L4 | 77.00 | 7.80 | 5.57 | 40.17 | 8.18 |
| L5 | 78.25 | 9.55 | 6.82 | 50.47 | 9.58 |
| L6 | 79.32 | 11.06 | 7.90 | 59.32 | 10.66 |

**Table S7.** Optimized parameters of the different systems considered in this study

| **Material** | **Refractive Index (RI)** | **Thickness (nm)** |
| --- | --- | --- |
| **Sys_1_** | | |
| BK7 (P) | 1.5151 | --- |
| Cu | 0.056253 + 4.2760 | 55.0 |
| **Sys_2_** | | |
| BK7 (P) | 1.5151 | --- |
| Cu | 0.056253 + 4.2760 | 55.0 |
| S_3_N_4_ (SN) | 2.0394 | 7.0 |
| **Sys_3_** | | |
| BK7 (P) | 1.5151 | --- |
| Cu | 0.056253 + 4.2760 | 40.0 |
| S_3_N_4_ (SN) | 2.0394 | 7.0 |
| Mxene | 2.38 + 1.33 i | 0.993*L (L=2) |
| **Sys_4_** | | |
| BK7 (P) | 1.5151 | --- |
| Cu | 0.056253 + 4.2760 | 45.0 |
| Mxene | 2.38 + 1.33 i | 0.993*L (L=1) |
| S_3_N_4_ (SN) | 2.0394 | 7.0 |

**Table S8.** Refractive index values for the different cancer types. The values of RI are reported before (normal) and after the cancer presence

| **Cancer Type** | **RI Normal** | **RI Cancer Presence** | $\boldsymbol{\Delta n}$ | **Ref.** |
| --- | --- | --- | --- | --- |
| Skin (Basal) | 1.360 | 1.380 | 0.020 | [35] |
| Cervical (HeLa) | 1.368 | 1.392 | 0.024 | [36] |
| Blood (Jurkat) | 1.376 | 1.390 | 0.014 | [37] |
| Adrenal (PC-12) | 1.381 | 1.395 | 0.014 | [38] |
| Breast T1 (MM-231) | 1.385 | 1.399 | 0.014 | [39] |
| Breast T2 (MCF-7) | 1.387 | 1.401 | 0.014 | [40] |

**Table S9.** Metrics of the different optimized systems for different cancer types

| **Cancer Type** | **SPR Peak position** | **Sensitivity Enhancement (%)** | $\boldsymbol{\Delta\theta}$ **(Deg)** | **Attenuation (%)** | **FWHM** |
| --- | --- | --- | --- | --- | --- |
| **Sys_1_** | | | | | |
| Skin | 72.93 | 3.89 | 2.73 | 1.15 | 0.72 |
| Cervical | 74.80 | 4.99 | 3.55 | 0.88 | 0.80 |
| Blood | 74.46 | 2.92 | 2.11 | 0.94 | 0.79 |
| Adrenal | 75.30 | 3.04 | 2.22 | 0.79 | 0.83 |
| Breast T1 | 76.00 | 3.14 | 2.31 | 0.65 | 0.86 |
| Breast T2 | 76.36 | 3.20 | 2.36 | 0.58 | 0.88 |
| **Sys_2_** | | | | | |
| Skin | 77.84 | 4.91 | 3.64 | 0.27 | 1.21 |
| Cervical | 80.65 | 6.75 | 5.10 | 0.04 | 1.45 |
| Blood | 80.13 | 4.01 | 3.09 | 0.00 | 1.40 |
| Adrenal | 81.49 | 4.41 | 3.44 | 0.27 | 1.53 |
| Breast T1 | 82.76 | 4.85 | 3.83 | 1.22 | 1.68 |
| Breast T2 | 83.48 | 5.14 | 4.08 | 2.28 | 1.78 |
| **Sys_3_** | | | | | |
| Skin | 80.06 | 5.16 | 3.93 | 8.71 | 6.51 |
| Cervical | 83.02 | 7.00 | 5.43 | 17.31 | 7.04 |
| Blood | 82.49 | 4.17 | 3.30 | 15.07 | 6.95 |
| Adrenal | 83.81 | 4.39 | 3.53 | 21.76 | 7.19 |
| Breast T1 | 84.78 | 4.37 | 3.55 | 30.56 | 7.41 |
| Breast T2 | 85.17 | 4.22 | 3.45 | 36.42 | 7.53 |
| **Sys_4_** | | | | | |
| Skin | 79.04 | 5.09 | 3.83 | 3.25 | 3.95 |
| Cervical | 82.06 | 7.10 | 5.44 | 7.99 | 4.58 |
| Blood | 81.50 | 4.23 | 3.31 | 6.70 | 4.45 |
| Adrenal | 82.98 | 4.68 | 3.71 | 10.69 | 4.79 |
| Breast T1 | 84.34 | 5.15 | 4.13 | 16.90 | 5.12 |
| Breast T2 | 85.07 | 5.41 | 4.36 | 22.08 | 5.32 |

**Table S10.** Performance metrics of the optimized SPR biosensors for different cancer types

| **Cancer Type** | ***S* (**$\boldsymbol{^{\circ}/RIU}$**)** | **DA** | **QF (*RIU*^-1^)** | **FoM (*RIU*^-1^)** | **LoD (10^-5^)** | **CSF** |
| --- | --- | --- | --- | --- | --- | --- |
| **Sys_1_** | | | | | | |
| Skin | 136.87 | 3.79 | 189.73 | 187.54 | 3.65 | 182.09 |
| Cervical | 148.17 | 4.40 | 183.39 | 181.76 | 3.37 | 175.53 |
| Blood | 151.33 | 2.67 | 191.05 | 189.25 | 3.30 | 182.87 |
| Adrenal | 158.92 | 2.67 | 190.78 | 189.27 | 3.14 | 182.65 |
| Breast T1 | 165.62 | 2.66 | 190.55 | 189.31 | 3.01 | 182.54 |
| Breast T2 | 169.19 | 2.62 | 190.41 | 189.30 | 2.95 | 182.48 |
| **Sys_2_** | | | | | | |
| Skin | 182.18 | 2.98 | 149.37 | 148.96 | 2.74 | 145.07 |
| Cervical | 212.76 | 3.51 | 146.45 | 146.39 | 2.35 | 141.89 |
| Blood | 220.98 | 2.20 | 157.36 | 157.36 | 2.26 | 152.61 |
| Adrenal | 245.98 | 2.23 | 159.97 | 159.54 | 2.03 | 154.51 |
| Breast T1 | 273.66 | 2.27 | 162.25 | 160.27 | 1.82 | 155.03 |
| Breast T2 | 291.96 | 2.28 | 163.38 | 159.65 | 1.71 | 154.32 |
| **Sys_3_** | | | | | | |
| Skin | 196.56 | 0.60 | 30.19 | 27.56 | 2.54 | 26.00 |
| Cervical | 226.56 | 0.77 | 32.15 | 26.58 | 2.20 | 24.39 |
| Blood | 236.16 | 0.47 | 33.97 | 28.85 | 2.11 | 26.60 |
| Adrenal | 252.23 | 0.49 | 35.04 | 27.42 | 1.98 | 24.94 |
| Breast T1 | 254.01 | 0.47 | 34.26 | 23.78 | 1.96 | 21.26 |
| Breast T2 | 246.42 | 0.45 | 32.68 | 20.78 | 2.02 | 18.33 |
| **Sys_4_** | | | | | | |
| Skin | 191.56 | 0.96 | 48.48 | 46.90 | 2.61 | 44.99 |
| Cervical | 226.82 | 1.18 | 49.52 | 45.56 | 2.20 | 43.06 |
| Blood | 236.60 | 0.74 | 53.11 | 49.55 | 2.11 | 46.94 |
| Adrenal | 265.17 | 0.77 | 55.34 | 49.42 | 1.88 | 49.53 |
| Breast T1 | 295.53 | 0.80 | 57.65 | 47.90 | 1.69 | 44.78 |
| Breast T2 | 312.05 | 0.82 | 58.62 | 45.67 | 1.60 | 42.44 |
